# Lipid droplet-mitochondria coupling via perilipin 5 augments respiratory capacity but is dispensable for FA oxidation

**DOI:** 10.1016/j.jlr.2022.100172

**Published:** 2022-01-21

**Authors:** Benedikt Kien, Stephanie Kolleritsch, Natalia Kunowska, Christoph Heier, Gabriel Chalhoub, Anna Tilp, Heimo Wolinski, Ulrich Stelzl, Guenter Haemmerle

**Affiliations:** 1 Institute of Molecular Biosciences, University of Graz, Graz, Austria; 2 Department of Pharmaceutical Chemistry, Institute of Pharmaceutical Sciences, University of Graz, Graz, Austria; 3 BioTechMed-Graz, University of Graz, Graz University of Technology and Medical University of Graz, Graz, Austria; 4 Field of Excellence BioHealth, University of Graz, Graz, Austria

**Keywords:** PLIN5, lipid droplets, lipolysis, mitochondrial respiration, FA oxidation, lipid droplet-mitochondria coupling, adipose-triglyceride lipase, comparative gene identification-58, lipotoxicity, cardiovascular disease, β-Gal, β-galactosidase, ASM, acid-soluble metabolite, ATGL, adipose triglyceride lipase, BAT, brown adipose tissue, CGI-58, comparative gene identification-58, DR, Deep Red, ETC, electron transport chain, EYFP, enhanced yellow fluorescent protein, IBMX, 3-isobutyl-1-methylxanthine, IP, immunoprecipitation, LD, lipid droplet, LDMC, LD-mitochondria coupling, OA, oleic acid, OCR, oxygen consumption rate, OXPHOS, oxidative phosphorylation, PA, palmitic acid, PGC-1α, peroxisome proliferator-activated receptor γ coactivator 1 alpha, PLIN5, perilipin 5, PKA, protein kinase A, TG, triacylglycerol

## Abstract

Disturbances in lipid homeostasis can cause mitochondrial dysfunction and lipotoxicity. Perilipin 5 (PLIN5) decorates intracellular lipid droplets (LDs) in oxidative tissues and controls triacylglycerol (TG) turnover via its interactions with adipose triglyceride lipase and the adipose triglyceride lipase coactivator, comparative gene identification-58. Furthermore, PLIN5 anchors mitochondria to the LD membrane via the outermost part of the carboxyl terminus. However, the role of this LD-mitochondria coupling (LDMC) in cellular energy catabolism is less established. In this study, we investigated the impact of PLIN5-mediated LDMC in comparison to disrupted LDMC on cellular TG homeostasis, FA oxidation, mitochondrial respiration, and protein interaction. To do so, we established PLIN5 mutants deficient in LDMC whilst maintaining normal interactions with key lipolytic players. Radiotracer studies with cell lines stably overexpressing wild-type or truncated PLIN5 revealed that LDMC has no significant impact on FA esterification upon lipid loading or TG catabolism during stimulated lipolysis. Moreover, we demonstrated that LDMC exerts a minor if any role in mitochondrial FA oxidation. In contrast, LDMC significantly improved the mitochondrial respiratory capacity and metabolic flexibility of lipid-challenged cardiomyocytes, which was corroborated by LDMC-dependent interactions of PLIN5 with mitochondrial proteins involved in mitochondrial respiration, dynamics, and cristae organization. Taken together, this study suggests that PLIN5 preserves mitochondrial function by adjusting FA supply via the regulation of TG hydrolysis and that LDMC is a vital part of mitochondrial integrity.

Lipid droplets (LDs) are highly dynamic organelles found in most eukaryotic cell types and consist of a triacylglycerol (TG)-rich neutral lipid core that is enveloped by a phospholipid monolayer (1). Perilipin 5 (Plin5/PLIN5) is a prominent member of the PAT protein family of LD-located proteins abundantly expressed in highly oxidative tissues (2, 3, 4). PLIN5 plays a key role in regulating intracellular lipid homeostasis by recruiting hormone-sensitive lipase to LDs (5) and coordinating the interactions of adipose triglyceride lipase (ATGL) with the ATGL coactivator comparative gene identification-58 (CGI-58) in a protein kinase A (PKA)-dependent manner (6, 7, 8, 9). An increasing amount of evidence suggests that PLIN5 protects cells from oxidative damage via multiple mechanisms that go beyond sole regulation of lipolysis. In mice and humans, Plin5/PLIN5 deficiency is associated with increased reactive oxygen species formation, impaired mitochondrial function, and cardiac myopathy, particularly upon ageing and following myocardial stress (10, 11, 12, 13, 14). In contrast, heart-specific Plin5 overexpression is compatible with normal life span, despite severe cardiac steatosis (6). In addition, PLIN5 has been shown to counteract oxidative stress and lipotoxicity in HepG2 cells and pancreatic β-cells (15, 16). These findings may in part be explained by the recently described role of PLIN5 in the nucleus, where the protein acts as a transcriptional modulator altering the expression of genes involved in mitochondrial function, reactive oxygen species defense, inflammation, and autophagy (17, 18, 19). Nonetheless, the cytoprotective effects of PLIN5 may involve other currently unknown functions of PLIN5.

Among mammalian PAT proteins, PLIN5 displays the unique characteristic of tightly anchoring mitochondria to the LD membrane via its last 20 amino acids (20, 21). This LD-mitochondria coupling (LDMC) has also been observed in vivo in tissues with high PLIN5 expression levels, such as brown adipose tissue (BAT) and heart (22, 23, 24). Previous studies proposed that LDMC enhances FA flux from LDs into mitochondria, thereby ensuring efficient β-oxidation (20, 24, 25, 26). In accordance with this model, PLIN5 is specifically enriched at the LD-mitochondria interface, especially during β3-adrenergic-stimulated conditions (24, 27). Moreover, LDMC significantly increases in cardiomyocytes upon fasting or β3-adrenergic stimulation (24, 28), whereas reduced LDMC via siRNA-mediated ablation of synaptosomal-associated protein 23 expression decreases mitochondrial FA oxidation (29). Despite these findings, the view that LDMC augments β-oxidation remains elusive, and a recent study suggests that LD-tethered mitochondria rely on pyruvate oxidation rather than on FA oxidation (23). In the present study, we examined the role of LDMC in intracellular lipid metabolism by employing carefully designed PLIN5 mutant variants specifically lacking interaction with mitochondria, while maintaining normal regulation of lipolysis. We demonstrate that PLIN5-mediated LDMC has no major impact on TG turnover or β-oxidation but significantly improves mitochondrial respiratory capacity and metabolic flexibility.

## Materials and methods

### Cell culture and transfection

Human embryonic kidney-293T cells (catalog no.: CRL-3216; ATCC) or COS-7 cells (catalog no.: CRL-1651; ATCC) were cultured in DMEM containing 4.5 g/l glucose, 10% FBS, 100 units/ml penicillin, and 100 μg/ml streptomycin. AML12 hepatocytes (catalog no.: CRL-2254; ATCC) were maintained in a 1:1 mixture of DMEM and Ham's F12 medium (2.5 mM l-glutamine and 15 mM Hepes) supplemented with 10% FBS, 100 units/ml penicillin, 100 μg/ml streptomycin, 0.005 mg/ml insulin, 0.005 mg/ml transferrin, 5 ng/ml selenium, and 40 ng/ml dexamethasone. AC16 cardiomyocytes (catalog no.: SSC109; Sigma-Aldrich) were cultivated in a 1:1 mixture of DMEM and Ham's F12 medium (2.5 mM l-glutamine and 15 mM Hepes) containing 12.5% FBS, 100 units/ml penicillin, and 100 μg/ml streptomycin. All cell lines were maintained in a standard humidified 7% CO_2_ atmosphere at 37°C. For experiments, cells were seeded in cell culture dishes or 25 cm^2^ cell culture flasks that were precoated with PBS containing 0.1% gelatin (catalog no.: G1393; Sigma-Aldrich). For transient protein expression, cells were transfected with plasmid DNA using Metafectene (catalog no.: T020; Biontex GmbH) according to the manufacturer's instructions. In indicated cases, cells were treated with oleic acid (OA) and/or palmitic acid (PA) conjugated to BSA (essentially FA-free; catalog no.: A6003; Sigma-Aldrich) at a molar FA-BSA ratio of 3:1.

### Plasmids, cloning of recombinant proteins, and site-directed mutagenesis

Complementary DNAs encoding murine *Plin5* (NM_025874.3), *Atgl* (NM_001163689.1), or *Cgi-58* (NM_026179.2) were cloned into the pcDNA4/HisMaxC expression vector (Invitrogen Life Technologies), as previously described (6, 30, 31). A pcDNA4/HisMax vector encoding *Escherichia coli* β-galactosidase (β-Gal) was provided by the manufacturer (Invitrogen Life Technologies). The detailed procedures for generation of pLVX-IRES-PURO (Clontech) constructs encoding recombinant proteins with an N-terminal FLAG tag as well as pEYFP-C1 (Takara Bio USA, Inc) constructs encoding target proteins with an N-terminal enhanced yellow fluorescent protein (EYFP) tag are described in the supplemental data section.

### Coimmunoprecipitation experiments

Human embryonic kidney-293T cells were cotransfected with expression vectors encoding FLAG-tagged- as well as Xpress-tagged recombinant proteins using Metafectene. Subsequently, the cells were lysed in ice-cold buffer A (50 mM Tris-HCl [pH 7.4], 150 mM NaCl, 1 mM EDTA, 1% [v/v] NP-40, 20 μg/ml leupeptine, 2 μg/ml antipain, and 1 μg/ml pepstatin). The FLAG-tagged proteins were precipitated from postnuclear supernatants using Anti-Flag M2 affinity gel (catalog no.: A2220; Merck Milipore). Copurifying proteins were identified by immunoblot analyses using adequate antibodies. Detailed procedures can be found in the supplemental data section.

### Generation of lentiviral particles and establishment of stable cell lines

Procedures are described in the supplemental data section.

### Confocal live cell microscopy

Cells were seeded in 8-well chambers mounted onto cover slips (catalog no.: 94.6170.802; Sarstedt). If required, cells were transfected with plasmid DNA encoding EYFP-fused target proteins using Metafectene. To induce PKA activity and stimulate lipolysis, cells were cultured in DMEM (1 g/l glucose) containing 20 μM forskolin (catalog no.: F6886; Sigma-Aldrich) and 500 μM 3-isobutyl-1-methylxanthine (IBMX; catalog no.: I5879; Sigma-Aldrich). For staining of LDs, cells were cultured in the presence of 2 μM BODIPY FL C_12_ (catalog no.: D3822; Green C12; Invitrogen) overnight. Subsequently, the cells were washed with PBS and cultured in fresh medium for 1 h to allow incorporation of residual Green C12 into TG. Prior to live cell imaging, mitochondria were stained by incubating the cells in medium containing 50 nM MitoTracker Deep Red (DR) FM (catalog no.: M22426; Invitrogen) for 30 min. Thereafter, the culture medium was renewed, and the cells were subjected to confocal laser scanning microscopy using a Leica SP8 confocal microscope (Leica Microsystems, Inc) equipped with a Leica HCX PL APO 63× 1.4 numerical aperture oil immersion objective. EYFP or Green C12 fluorescence was exited at 488 nm, and emission was detected between 500 and 550 nm. MitoTracker DR was excited at 633 nm, and emission was detected between 660 and 750 nm. The acquired images were adjusted for brightness and contrast using ImageJ software (the National Institutes of Health), and the fluorescence signals were pseudocolored in green (EYFP/Green C12) and magenta (MitoTracker DR). Zoomed insets were generated using an ImageJ macro tool kindly provided by Gilles Carpentier (Paris-East Créteil University, Paris, France), available under the link: http://image.bio.methods.free.fr/ImageJ/?Zoom-in-Images-and-Stacks&lang=en&artpage=2-2 (as of December 2021). The mitochondrial recruitment to LDs was quantified using ImageJ software, as described in the supplemental data section.

### Endogenous TG levels of COS-7 fibroblasts

COS-7 cells were seeded in 100 mm cell culture dishes and cultivated under standard conditions for 24 h. The cells were washed three times with PBS, harvested using a cell scraper, and pelleted in 2 ml reaction vials by centrifugation at 320 *g* and 4°C for 3 min. The cellular lipids were extracted twice in hexane/isopropanol (3/2; v/v) for 10 min at room temperature, followed by drying of the lipids under a stream of nitrogen. The residual cellular proteins were dried for 1 h at 40°C on a heat block, with the caps of the reaction vials left open. Subsequently, the proteins were solubilized in 0.5 ml of a solution containing 0.3 N NaOH and 0.1% (w/v) SDS by shaking on a heat block at 60°C and 900 rpm for 2 h. Protein concentrations were determined using Pierce BCA reagent according to the manufacturer's instructions. Lipids corresponding to equal amounts of cellular protein (600 μg) were reconstituted in CHCl_3_ and spotted onto a TLC silica plate (TLC Silica gel 60 aluminum sheets, 20 × 20 cm, Merck no.: 1.05553.0001, predried for 1 h at 60°C). The plate was developed using hexane/diethyl ether/acetic acid (70/29/1; v/v/v) as solvent system. To visualize the lipid bands, the plate was dipped into charring solution (ethanol 25%, H_3_PO_4_ 10%, CuSO_4_ 5%), dried, and incubated for 20 min at 140°C. Following imaging via the Chemidoc Touch Imaging System (Bio-Rad), the intensities of TG-standard corresponding bands were quantified using ImageJ software.

### Pulse-chase experiments

Cells were seeded in 6-well cell culture plates, followed by 20–24 h of incubation in medium containing either 0.4 mM OA-BSA, or a mixture of 0.2 mM OA-BSA and 0.2 mM PA-BSA, using 1 μCi/ml [9,10-^3^H]-OA-BSA (catalog no,: ART0198; Hartmann Analytic) or 0.4 μCi/ml [1-^14^C]-PA-BSA (catalog no.: MC121; Hartmann Analytic) as tracer (pulse). Subsequently, cells were washed three times with PBS. To stimulate lipolysis, pulsed cells were incubated in serum-free DMEM (1 g/l glucose) containing 3% (w/v) FA-free BSA and 20 μM forskolin (chase). Following washing with PBS, cellular lipids were extracted twice in hexane/isopropanol (3/2; v/v) for 10 min at room temperature, dried under a stream of nitrogen, reconstituted in CHCl_3_, and developed by TLC using hexane/diethyl ether/acetic acid (70/29/1; v/v/v) as solvent system. Lipids were visualized by iodine vapor staining. TG-standard corresponding bands were excised, submerged in scintillation cocktail (Rotiszint; catalog no.: 0016.3; Carl Roth GmbH) and subjected to scintillation counting. Remaining cellular proteins were solubilized in a solution containing 0.3 N NaOH and 0.1% (w/v) SDS by shaking for 6 h at room temperature, and protein concentrations were determined using Pierce BCA reagent. To determine total cellular FA incorporation, culture medium aliquots were collected prior to, as well as after the pulse period, followed by centrifugation at 10,000 rpm for 10 min and analysis of supernatants (200 μl) by liquid scintillation counting.

### Cellular FA release in the presence or the absence of triacsin C

Cells were seeded in 6-well cell culture plates, followed by 20 h of cultivation in medium containing 0.4 mM OA-BSA and 1 μCi/ml [9,10-^3^H]-OA-BSA as tracer (pulse). Incorporation of radioactivity into TG and total cellular label incorporation during the pulse period were determined as described previously. Next, the cells were washed three times with PBS and preincubated for 1 h in complete medium containing either DMSO (vehicle) or 5 μM triacsin C (catalog no.: T4540; Merck Millipore). The cells were washed again with PBS, followed by 3.5 h of cultivation in serum-free DMEM (1 g/l glucose) containing 3% (w/v) FA-free BSA and 20 μM forskolin together with DMSO or 5 μM triacsin C (chase). To determine FA release, medium aliquots were collected at the end of the chase period and clarified by centrifugation at 10,000 rpm for 10 min, followed by liquid scintillation counting of supernatants (200 μl).

### FA oxidation of intact mammalian cells

Cells were seeded in 25 cm^2^ cell culture flasks. To quantify oxidation of exogenously added FAs, cells were cultured in DMEM (1 g/l glucose) containing 1% FBS and 0.5 mM l-carnitine for 24 h prior to the assay. Subsequently, the medium was changed to serum-free DMEM (1 g/l glucose) supplemented with 0.5 mM l-carnitine, 100 μM PA-BSA, and 0.4 μCi [1-^14^C]-PA-BSA as tracer. The flasks were immediately sealed with a rubber plug holding a center well that contained a piece of Whatman filter paper, which was soaked with 30 μl 5 N NaOH. Following 90 min of incubation at 37°C, 100 μl HClO_4_ was injected into the assay medium in the flasks, using a syringe, and the released CO_2_ was trapped in the filter paper for 2 h at 37°C. Thereafter, assay medium aliquots were collected and clarified by centrifugation at 10,000 rpm for 10 min. The production of radiolabeled CO_2_ and incompletely oxidized acid-soluble metabolites (ASMs) was quantified by liquid scintillation counting of the filter papers and assay medium supernatants (200 μl), respectively. Following extensive washing with PBS to remove precipitated BSA, the remaining cellular proteins were solubilized in 0.3 N NaOH and 0.1% (w/v) SDS by shaking for 6 h at room temperature. Protein content was determined using Pierce BCA reagent.

To analyze oxidation of LD-derived FAs, AC16 cells were cultured in complete medium containing 0.5 mM l-carnitine, 0.2 mM OA-BSA, 0.2 mM PA-BSA, and 0.4 μCi/ml [1-^14^C]-PA-BSA as tracer for 16 h. Subsequently, the cells were washed three times with PBS, and the medium was changed to serum-free DMEM (1 g/l glucose) supplemented with 0.5 mM l-carnitine and 20 μM forskolin. The production of radiolabeled CO_2_ and the generation of ASM were determined as described previously, with the exception that the cells were incubated for 2 h at 37°C prior to HClO_4_ injection.

### MS

Immunoprecipitation (IP)-purification proteomics of AC16 cardiomyocytes were performed using diaPASEF MS. Detailed procedures are described in the supplemental data section. The MS proteomics data have been deposited to the ProteomeXchange Consortium via the PRIDE (32) partner repository with the dataset identifier PXD028541.

### Seahorse XF respirometry of living AC16 cells

The cellular oxygen consumption rate (OCR) as well as the extracellular acidification rate of AC16 cells upon sequential injection of 20 μM forskolin, 1 μM oligomycin A (catalog no.: 75351; Sigma-Aldrich), 1 μM carbonyl cyanide 4-(trifluoromethoxy)phenylhydrazone (catalog no.: C2920; Sigma-Aldrich) and 1 μM antimycin A (catalog no.: A8674; Sigma-Aldrich) (final concentrations) were determined using the Seahorse XFe96 analyzer (Agilent Technologies). Assay medium consisted of DMEM (catalog no.: D5030; Gibco) containing 5 mM glucose and 2 mM GlutaMAX, adjusted to pH 7.4 prior to the measurement. For detailed procedures, see the supplemental data section.

### Subcellular fractionation

Mitochondria-enriched fractions as well as nuclear fractions were obtained by differential centrifugation. Detailed methods can be found in the supplemental data section.

### Quantification of mitochondrial DNA content and gene expression analyses

Mitochondrial DNA content as well as *PDK4* gene expression levels of AC16 cells were determined by quantitative PCR. For detailed procedures, see the supplemental data section.

### Immunoblot analyses

Immunoblot analyses were performed according to standard protocols. Further details including the used antibodies are described in the supplemental data section.

## Results

### PLIN5 promotes LDMC via its C-terminal region under basal and PKA-stimulated conditions

**Fig. 1:**
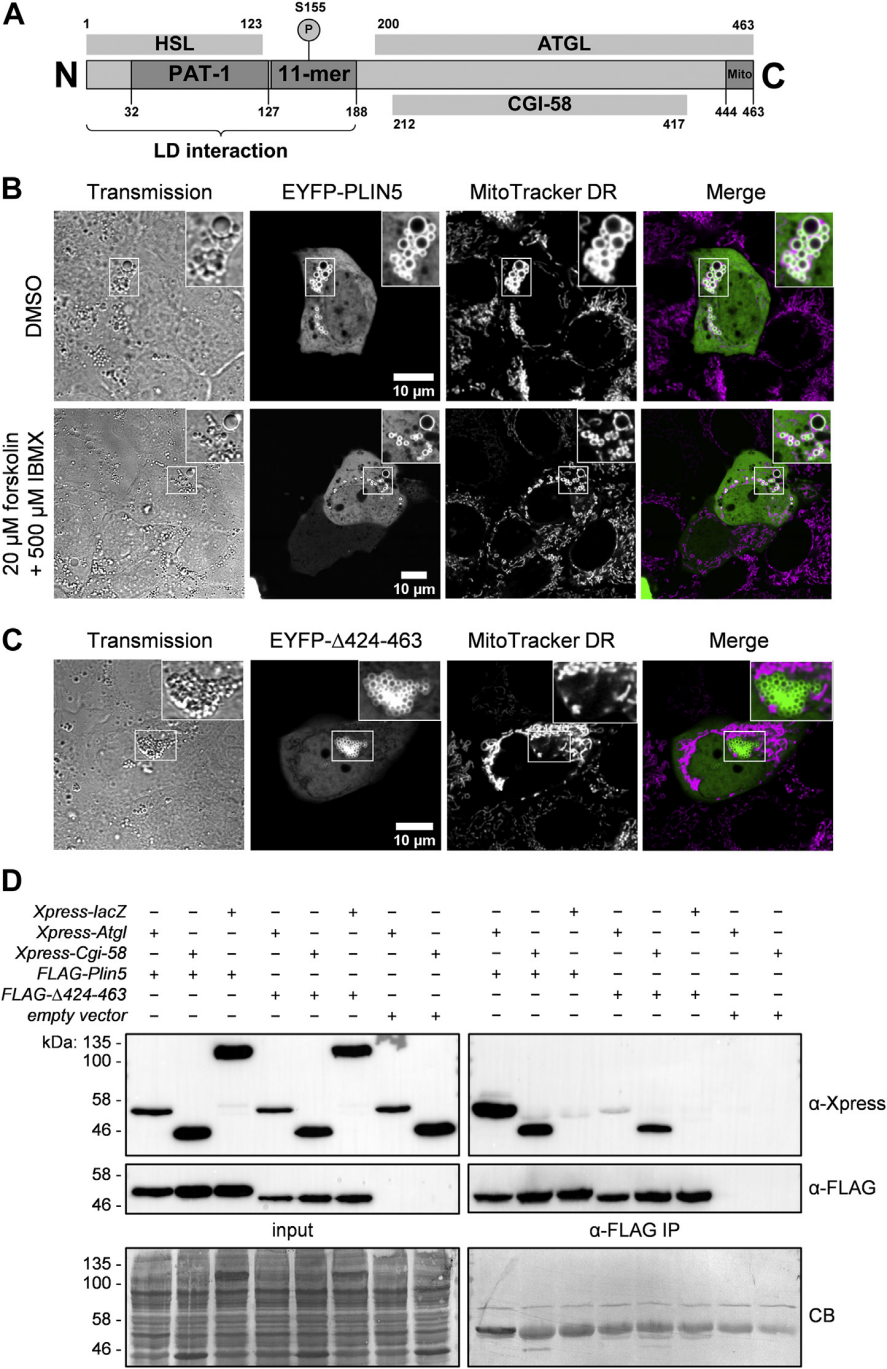
The carboxyl-terminal region of PLIN5 promotes LDMC under basal and forskolin-stimulated conditions. A: Currently established domain architecture of murine PLIN5. The crucial regions for LD localization, PKA phosphorylation, as well as interaction with HSL, ATGL, CGI-58, and mitochondria are indicated. B: AML12 hepatocytes were transfected with plasmid DNA encoding *EYFP-Plin5* and cultured for 24 h. Subsequently, the cells were treated for 2 h with DMSO (vehicle) or forskolin together with IBMX to stimulate PKA activity and consequently lipolysis. Mitochondria were stained using MitoTracker DR, followed by confocal microscopy analyses. C: Live cell imaging of AML12 cells transiently expressing EYFP-Plin5(Δ424–463). The mutant protein displays normal LD localization but lacks interaction with mitochondria. D: FLAG-tagged PLIN5(Δ424–463) exhibits substantially reduced interaction with Xpress-tagged ATGL. Recombinant proteins were coexpressed in human embryonic kidney-293T cells and detected in postnuclear supernatants (input) and anti-FLAG IPs by immunoblot analyses using the respective tag-specific antibodies. Equal protein loading was verified by Coomassie blue (CB) staining. Insets, 2× magnification.

PLIN5 plays a major role in the regulation of lipolysis by concerting the interactions of ATGL with the ATGL coactivator CGI-58 in a PKA-dependent manner (6, 7, 8, 9) (Fig. 1A). Moreover, PLIN5 has been reported to mediate LDMC via its last 20 C-terminal amino acids, comprising an evolutionary conserved sequence with a distinct charge distribution (20). However, the metabolic consequences of PLIN5-mediated LDMC are incompletely understood and require further examination.

First, we investigated the impact of PKA activation on PLIN5-mediated LDMC. Hence, we transiently expressed EYFP-tagged PLIN5 in AML12 hepatocytes, followed by treatment with either DMSO (vehicle) or forskolin together with IBMX, to induce PKA activity. Subsequently, mitochondria were stained with MitoTracker DR, and cells were subjected to confocal live cell microscopy. Figure 1B depicts representative images of AML12 cells in the basal state and upon β-adrenergic stimulation. In the basal state, most mitochondria were tightly attached to the surface of PLIN5-enriched LDs, contrasting the typical reticular appearance of the mitochondrial network (Fig. 1B, upper panel). Moreover, we observed that LDMC persisted upon forskolin/IBMX treatment (Fig. 1B, lower panel), which is in accordance with a recent study demonstrating that fasting or β3-adrenergic stimulation mildly increases LDMC in cardiac tissue of mice (24). Immunoblot analysis using an antiphospho-PKA substrate antibody as a measure of β-adrenergic stimulation confirmed forskolin-induced PKA substrate phosphorylation (supplemental Fig. S1).

Next, we aimed to establish suitable cell models to address the specific impact of LDMC on cellular TG homeostasis, FA oxidation, and mitochondrial respiration. In a previous study, expression of a PLIN5 mutant deficient for the last 65 C-terminal amino acids (Δ399–463) was used to study the impact of LDMC on lipid metabolism (23). There, the authors observed reduced TG synthesis in brown adipocytes and pancreatic beta cells overexpressing PLIN5(Δ399–463), compared with cells overexpressing wild-type PLIN5. We generated a similar EYFP-PLIN5(Δ424–463) mutant (supplemental Fig. S2) that also lacked interaction with mitochondria, while maintaining normal LD surface localization (Fig. 1C). However, co-IP analysis revealed substantially reduced protein interaction of FLAG-tagged PLIN5(Δ424–463) with Xpress-tagged ATGL, and even the interaction with Xpress-CGI-58 appeared to be moderately reduced (Fig. 1D). This finding is in line with a study by Granneman *et al.* (8), reporting that a PLIN5(Δ400–463) variant interacts with neither ATGL nor CGI-58. These data suggest that both PLIN5(Δ399–463) and PLIN5(Δ424–463) may interfere with physiological regulation of lipolysis because of altered interaction with lipolytic key players.

### PLIN5 truncation variants reveal crucial regions involved in PLIN5 interactions with ATGL and mitochondria

**Fig. 2:**
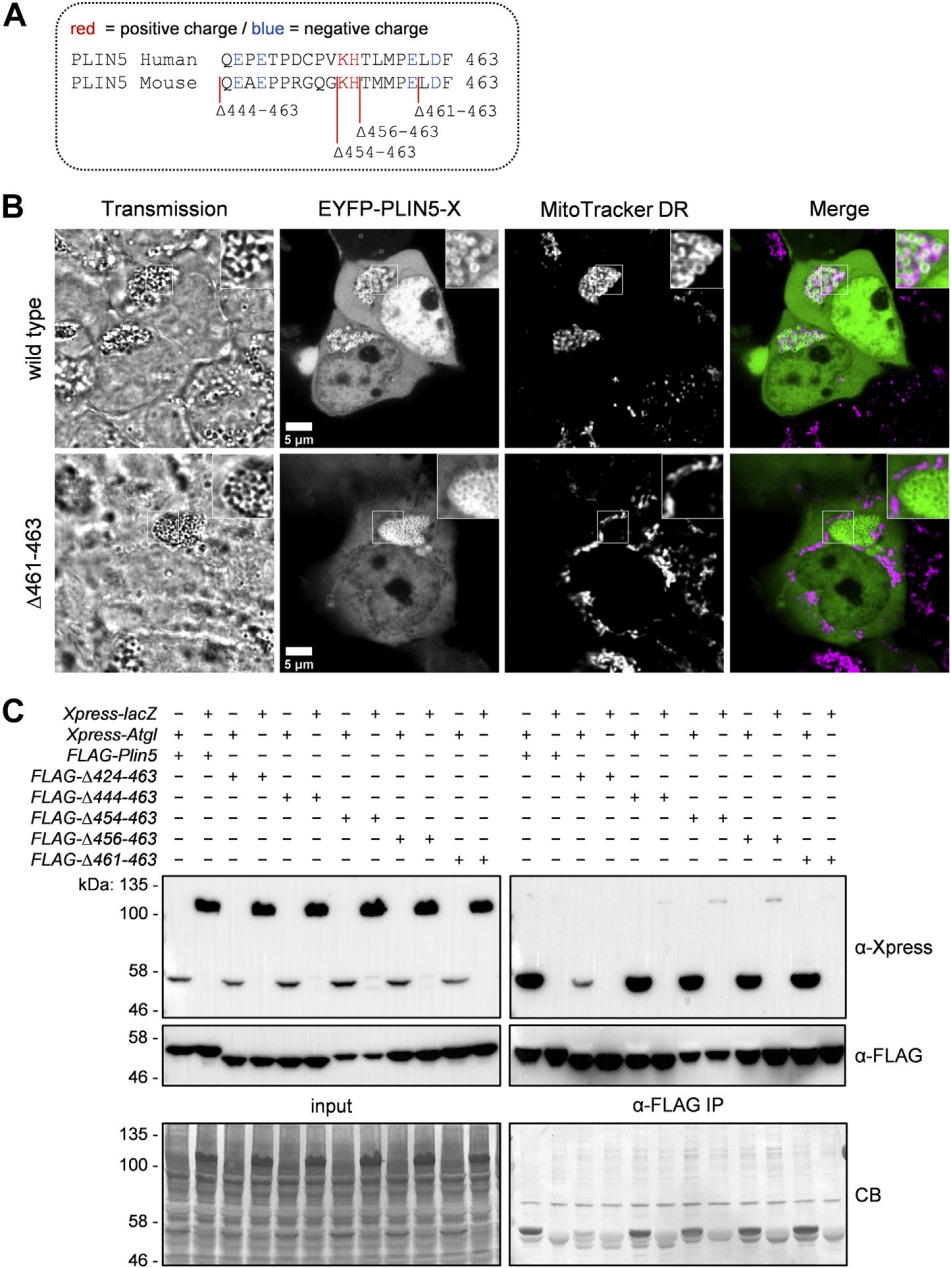
Generation of truncated PLIN5 variants specifically lacking interaction with mitochondria, while maintaining normal interaction with ATGL. A: Protein sequence alignment of the conserved carboxyl terminus of human and murine PLIN5. Positive or negative charged polar amino acid residues are highlighted in red and blue, respectively. Vector constructs encoding C-terminally truncated PLIN5 mutants were generated by inserting stop codons into the *Plin5* CDS via site-directed mutagenesis. B: Truncation of the C-terminal Leu-Asp-Phe (LDF) sequence of PLIN5 is sufficient to disrupt LDMC. Human embryonic kidney-293T (HEK-293T) cells transiently expressing EYFP-tagged wild type or mutant PLIN5 were cultured in medium containing oleic acid to promote LD formation. Mitochondria were stained using MitoTracker DR, and cells were analyzed by confocal microscopy. C: The region spanning amino acids 424–443 of murine PLIN5 is critical for robust protein interaction with ATGL. HEK-293T cells were cotransfected with plasmid DNA encoding recombinant proteins as indicated. Target proteins were detected in cell homogenates (input) and anti-FLAG IPs by immunoblot analyses using adequate antibodies. Coomassie blue (CB) staining confirmed equal protein loading. Insets, 2× magnification.

Our findings prompted us to generate and overexpress mutant PLIN5 lacking different portions of the conserved C terminus (Fig. 2A and supplemental Fig. S2) and study their impact on LDMC. Similar to PLIN5(Δ424–463), all these truncation mutants lacked interaction with mitochondria (supplemental Fig. S3). Unexpectedly, even deletion of solely the last three amino acids of PLIN5 (PLIN5[Δ461–463]) was sufficient to disrupt LDMC (Fig. 2B), highlighting the importance of the conserved C-terminal charge distribution (20) for interaction with mitochondrial membranes and/or mitochondrial proteins. Importantly, all the generated PLIN5 mutants, including FLAG-PLIN5(Δ444–463), exhibited affinity for interaction with Xpress-ATGL similar to wild-type PLIN5 (Fig. 2C). These data indicate that a region spanning amino acids 424–443 of murine PLIN5 is critical for robust protein interaction with ATGL. Thus, we have generated PLIN5 mutants that specifically lack interaction with mitochondria, while maintaining normal interaction with lipolytic key players including ATGL and CGI-58. Hence, these mutant proteins are suitable to investigate the role of LDMC versus disrupted LDMC in cellular lipid metabolism.

### Abolishment of PLIN5-mediated LDMC does not alter intracellular TG turnover

**Fig. 3:**
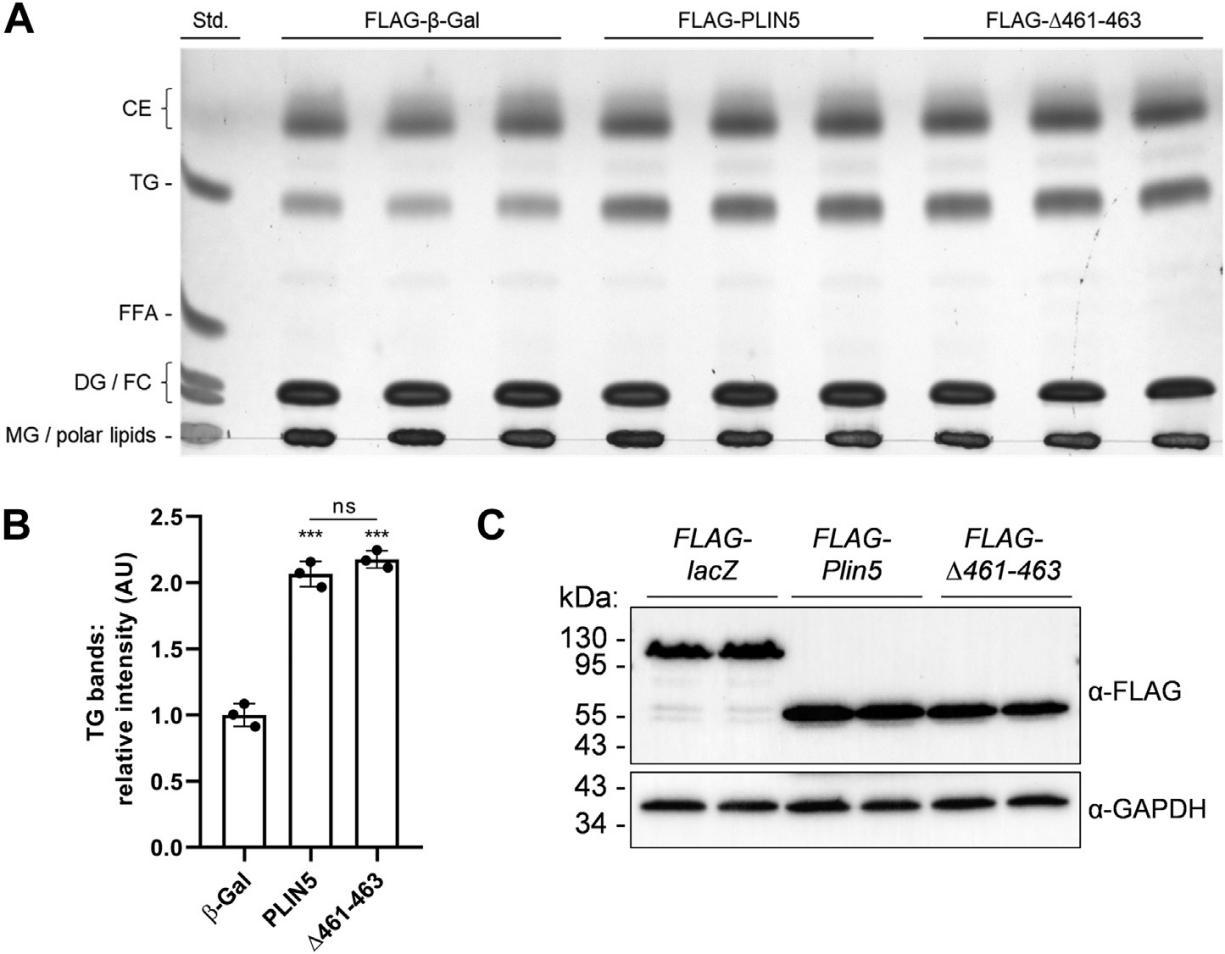
PLIN5(Δ461–463) overexpression similarly increased endogenous TG levels compared with PLIN5-overexpressing COS-7 fibroblasts. A: Cellular lipids from COS-7 cells stably expressing recombinant target proteins, as indicated, were extracted and separated by TLC using hexane/diethyl ether/acetic acid (70/29/1; v/v/v) as solvent system. Lipid bands were visualized by charring. B: Densitometric analysis of TG corresponding signal intensities in transgenic cells relative to the TG-standard determined by ImageJ software. C: Similar expression levels of the target proteins were verified by immunoblot analysis using an anti-FLAG epitope antibody and an anti-GAPDH antibody, respectively. Data are presented as mean ± SD (n = 3). Statistical significance was determined by unpaired Student’s *t*-test (ns = not significant, ∗∗∗*P* < 0.001 vs. β-Gal). CE, cholesteryl ester; DG, diacylglycerol; FC, free cholesterol; MG, monoacylglycerol.

Next, we established multiple cell lines stably overexpressing FLAG-tagged β-Gal (control), PLIN5, or PLIN5(Δ461–463) via lentiviral transduction. Puromycin selection ensured that the recombinant proteins were expressed in virtually every cell. Notably, stable overexpression of PLIN5(Δ461–463) abrogates LDMC in COS-7 (supplemental Fig. S4), AML12 (supplemental Fig. S5), and AC16 (supplemental Fig. S6) cell lines assessed by confocal live cell imaging. In all these cell lines, we could observe a significant reduction of LDMC upon overexpression of mutant PLIN5 merely lacking the last three amino acids at the C terminus (supplemental Fig. S7). Next, we investigated the impact of disrupted LDMC on endogenous TG homeostasis in COS-7 cells overexpressing PLIN5 or PLIN5(Δ461–463), respectively. Transgenic COS-7 cells were incubated in complete medium followed by lipid extraction and lipid separation by TLC. Lipids were visualized by charring (Fig. 3A) and quantified by densitometry analysis (Fig. 3B). In line with numerous studies, overexpression of PLIN5 raised endogenous TG levels (∼2-fold) compared with β-Gal-expressing control cells. Interestingly, overexpression of PLIN5(Δ461–463) increased TG levels to a similar extent compared with PLIN5 implicating that disruption of LDMC does not divergently impact TG homeostasis compared with cells with predominant LDMC. Western blot analysis revealed similar expression levels of the recombinant proteins (Fig. 3C).

**Fig. 4:**
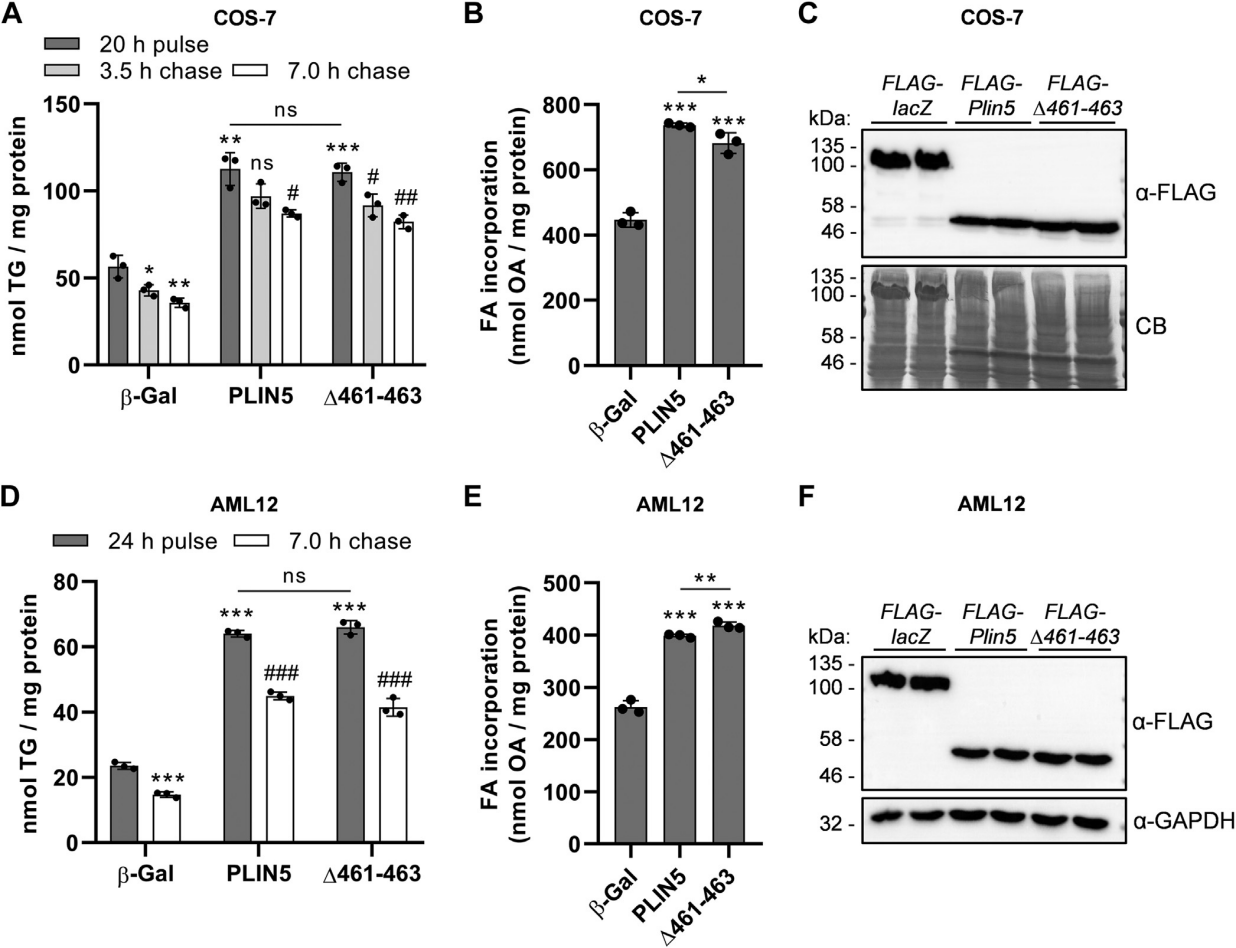
Disruption of LDMC upon PLIN5(Δ461–463) overexpression similarly interfered with TG homeostasis compared with PLIN5-mediated LDMC. COS-7 fibroblasts (A–C) or AML12 hepatocytes (D–F) were transduced with lentiviral particles encoding FLAG-tagged recombinant target proteins, as indicated, followed by puromycin selection. Cells were incubated in medium containing 0.4 mM BSA-conjugated OA for 20–24 h, using ^3^H-labeled OA-BSA as tracer (pulse). Subsequently, cells were cultured in serum-free DMEM (1 g/l glucose) containing 20 μM forskolin and 3% (w/v) FA-free BSA for the indicated time to induce lipolysis (chase). A, D: Total lipids were extracted and separated by TLC, and radioactivity in the TG-corresponding bands was determined by liquid scintillation counting. B, E: Radioactivity in culture medium supernatants prior to and after the pulse period was quantified to determine cellular FA incorporation. C, F: Expression levels of the target proteins were probed by immunoblot analyses, using an anti-FLAG-HRP antibody. Equal protein loading was verified by Coomassie blue (CB) staining or by detection of GAPDH. All data are presented as mean ± SD (n = 3). Statistical significance was determined by unpaired Student’s *t*-test (ns = not significant, ∗*P* < 0.05, ∗∗*P* < 0.01, ∗∗∗*P* < 0.001 vs. β-Gal pulse; #*P* < 0.05, ##*P* < 0.01, ###*P* < 0.001 vs. PLIN5 or Δ461–463 pulse).

To further study the impact of abolished LDMC on cellular lipid turnover, we subjected transgenic COS-7 fibroblasts (Fig. 4A, B) or AML12 hepatocytes (Fig. 4D, E) to pulse-chase experiments using radiolabeled OA as tracer. Expression levels of the recombinant proteins were comparable in the transgenic cell lines (Fig. 4C, F, respectively). As previously described (31, 33), upon 20–24 h of OA loading (pulse), ectopic expression of PLIN5 significantly increased OA esterification into TG by approximately 2- to 3-fold relative to control cells (Fig. 4A, D). In line with this finding, total cellular OA incorporation during the pulse period was significantly increased in PLIN5-expressing cells (Fig. 4B, E). However, we observed no significant differences between expression of wild type or mutant PLIN5 with regard to TG synthesis during the pulse or TG catabolism during forskolin-stimulated lipolysis (chase) (Fig. 4A, D). In accordance with comparable TG production, cellular OA uptake during the pulse was similarly increased in cells expressing wild type or mutant PLIN5, with only minor, yet significant differences (Fig. 4B, E). Notably, PLIN5-expressing COS-7 cells degraded only ∼24% of their TG during the 7 h of chase, compared with a mean TG degradation of ∼37% in β-Gal control cells. In contrast, the mean TG degradation of the AML-12 cell lines during the chase was similar (β-Gal: ∼38%; PLIN5: ∼30%; Δ461–463: ∼37%). This indicates a higher lipolytic capacity of AML12 hepatocytes upon PLIN5 overexpression relative to COS-7 fibroblasts. Taken together, these findings suggest that LDMC exerts no major impact on either FA esterification upon lipid loading or TG degradation during stimulated lipolysis in living cells.

**Fig. 5:**
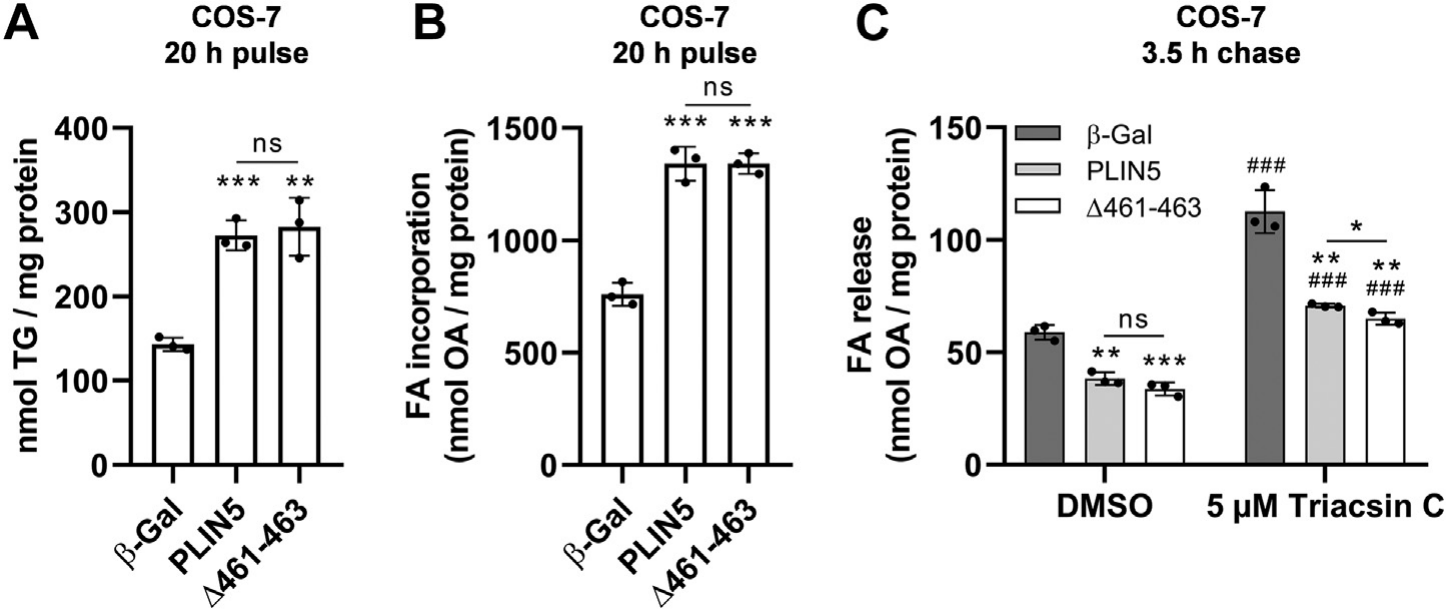
Abolished LDMC because of PLIN5(Δ461–463) overexpression similarly interfered with re-esterification and FA release compared with cells overexpressing PLIN5. Transgenic COS-7 cells were cultured in medium containing 0.4 mM OA-BSA for 20 h, using ^3^H-labeled OA-BSA as tracer (pulse). A: Incorporation of radioactivity into TG during the pulse period was determined by separation of lipid extracts via TLC, followed by excision and liquid scintillation counting of TG-standard corresponding bands. B: Total cellular label incorporation during the FA pulse was determined by scintillation counting of medium supernatants prior to and after the pulse period. C: Following the pulse period, the cells were preincubated for 1 h in complete medium containing DMSO (vehicle) or 5 μM triacsin C. Subsequently, the cells were cultured for 3.5 h in serum-free DMEM (1 g/l glucose) containing 20 μM forskolin and 3% (w/v) FA-free BSA together with either DMSO or 5 μM triacsin C (chase). The FA release into the medium was quantified by liquid scintillation counting. Data are presented as mean ± SD (n = 3). Statistical significance was determined by unpaired Student’s *t*-test (ns = not significant, ∗*P* < 0.05, ∗∗*P* < 0.01, ∗∗∗*P* < 0.001 vs. corresponding β-Gal conditions; ###*P* < 0.001 vs. corresponding DMSO conditions).

It is conceivable that the abrogated interaction of PLIN5(Δ461–463) with mitochondria (and consequently disrupted LDMC) could interfere with the re-esterification rates of LD-derived FAs during lipolysis. To address this assumption, we quantified the FA release from transgenic COS-7 cells into the culture medium upon forskolin-stimulated lipolysis, either in the presence or the absence of triacsin C. Therefore, we subjected the cells to a 20 h of OA pulse using ^3^H-labeled OA as tracer. In line with our previous findings, FA incorporation into TG (Fig. 5A) as well as total cellular label incorporation (Fig. 5B) were similarly increased in cells expressing wild type or mutant PLIN5 as compared with control cells. Following the pulse period, cells were cultured for 3.5 h in serum-free starvation medium containing forskolin together with DMSO (control) or triacsin C to inhibit re-esterification. Triacsin C treatment significantly increased the FA release into the medium of all tested cell lines relative to the DMSO condition (Fig. 5C). Compared with β-Gal control cells, FA release of PLIN5-overexpressing or PLIN5(Δ461–463)-overexpressing cells was comparably reduced albeit FA release was marginally (−8%) but significantly different among PLIN5 and PLIN5(Δ461–463)-overexpressing cells. Taken together, these findings suggest that overexpression of both PLIN5 or mutant PLIN5(Δ461–463) comparably impacts TG catabolism and re-esterification beyond the presence or the absence of LDMC.

### Disruption of LDMC moderately lowers β-oxidation rates of exogenously added or LD-derived FAs

**Fig. 6:**
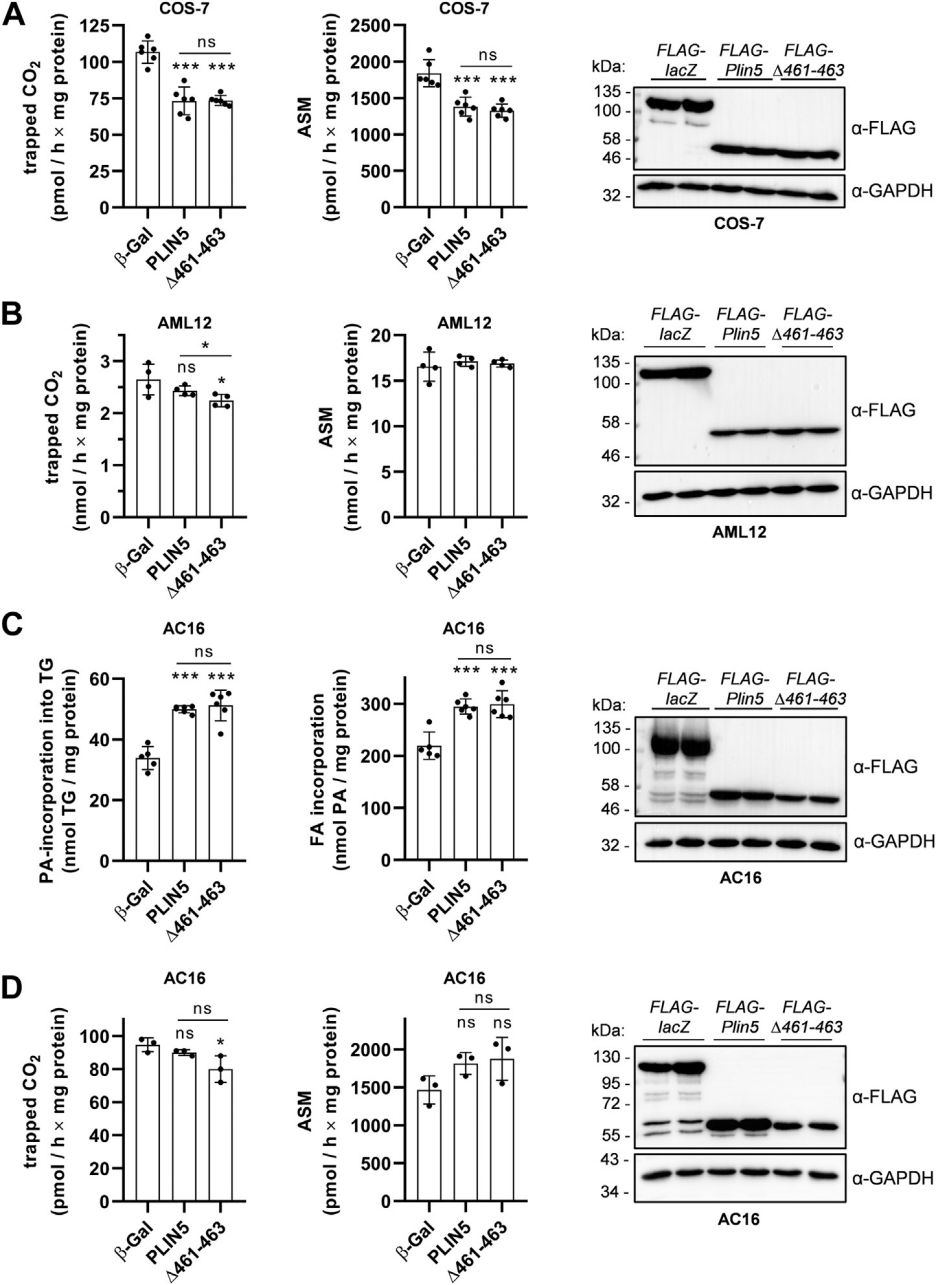
Disruption of LDMC has no major impact on β-oxidation rates of exogenously added or LD-derived FAs compared with cells with PLIN5-mediated LDMC. A, B: Acute β-oxidation rates of exogenously added PA-BSA of (A) COS-7 cells or (B) AML12 cells stably expressing recombinant target proteins as indicated. A, B, left panels: Released radiolabeled ^14^C-CO_2_ was trapped in NaOH-soaked filter paper and quantified by liquid scintillation counting. A, B, middle panels: The generation of PA-derived ^14^C-labeled ASMs was quantified by determining the radioactivity in culture medium supernatants. A, B, right panels: Immunoblot analyses verified similar expression levels of target proteins during the assays. C: Transgenic AC16 cells were pulsed with a mixture of 0.2 mM OA-BSA and 0.2 mM PA-BSA for 16 h, using ^14^C-labeled PA-BSA as tracer. PA-incorporation into TG (C, left panel) and total cellular PA uptake during the pulse (C, middle panel) were quantified by scintillation counting of TG fractions and culture medium supernatants, respectively. D: Oxidation rates of LD-derived FAs. Transgenic AC16 cells were pulsed as in (C), followed by stimulation of lipolysis via serum starvation and forskolin treatment. Released ^14^C-CO_2_ (D, left panel) and generated PA-derived ASM (D, middle panel) were quantified as mentioned. C, D, right panels: Overexpression of target proteins during the experiments was confirmed by immunoblot analyses. All data are presented as mean ± SD (A: n = 6; B: n = 4; C: n = 5–6; and D: n = 3). Statistical significance was determined by unpaired Student’s *t*-test (ns = not significant, ∗*P* < 0.05, ∗∗∗*P* < 0.001 vs. β-Gal).

Close proximity of LDs and mitochondria has been proposed to spatially connect TG catabolism and FA release to mitochondrial FA uptake and β-oxidation. The marked divergences in the spatial distribution of LDs and mitochondria upon overexpression of PLIN5 or PLIN5(Δ461–463) prompted us to examine the impact of increased versus abolished LDMC on mitochondrial FA oxidation. Therefore, we measured FA oxidation in transgenic COS-7 cells incubated with serum-free low glucose DMEM supplemented with l-carnitine, 100 μm PA, and ^14^C-labeled PA followed by the quantification of ^14^C-CO_2_ release as a measure of FA oxidation and the generation of incompletely oxidized FA designated as ASMs. Notably, stable overexpression of both PLIN5 or PLIN5(Δ461–463) similarly reduced CO_2_ release (Fig. 6A, left; mean CO_2_ −31%) and ASM production (Fig. 6A, middle; mean ASM −25% to −28%) despite the marked divergences in LDMC. Next, we investigated PA oxidation in PLIN5-transgenic and PLIN5(Δ461–463)-transgenic AML12 cell lines. Interestingly, CO_2_ release was marginally (−4%) but significantly reduced in PLIN5(Δ461–463) compared with PLIN5 transgenic AML12 cells (Fig. 6B, left), whereas ASM levels were comparable to control cells (Fig. 6B, middle). Of note, AML12 cells displayed significantly higher CO_2_ production in the nanomolar range, compared with the picomolar range in the COS-7 fibroblasts, suggesting an overall higher oxidative capacity of the hepatocyte cell line. Immunoblot analyses revealed comparable expression levels of the recombinant proteins (Fig. 6A, B, right). Together, these findings suggest that LDMC does not significantly enhance mitochondrial FA oxidation.

It is conceivable that exogenously added FAs can bypass the intracellular TG pool and thus are preferentially oxidized in mitochondria (34). Hence, we also investigated the impact of LD-derived FAs on mitochondrial FA oxidation in the presence and absence of LDMC. Therefore, we chose the human AC16 cardiomyocyte cell line as an additional oxidative cell type model. As with our previous models, we stably overexpressed FLAG-tagged β-Gal, PLIN5, or PLIN5(Δ461–463) in AC16 cells. Confocal microscopy analysis also revealed abolished LDMC upon stable overexpression of PLIN5(Δ461–463) in AC16 cells (supplemental Figs. S6 and S7), which is in line with disrupted LDMC in transgenic COS-7 and AML-12 cells, respectively. Next, we subjected the cells to a 16 h pulse with a mixture of OA and PA at a 1:1 M ratio to promote LD formation. Using ^14^C-labled PA as tracer, we observed ∼50% increased incorporation of PA into TG in the PLIN5/PLIN5(Δ461–463)-overexpressing AC16 cells (Fig. 6C, left), which was mirrored by a significant increase in total label incorporation compared with β-Gal control cells (Fig. 6C, middle). Despite slightly reduced PLIN5(Δ461–463) protein expression levels (Fig. 6C, right), we observed almost identical incorporation of radioactivity into TG in cells expressing PLIN5 compared with PLIN5(Δ461–463), further supporting the notion that LDMC does not enhance FA esterification. In the following, we subjected 16-h pulsed AC16 cells to 2 h of forskolin treatment and serum starvation to induce lipolysis. In line with our previous results, CO_2_ release was slightly reduced in PLIN5 and PLIN5(Δ461–463) transgenic AC16 cells (Fig. 6D, left) albeit differences for PLIN5 compared with β-Gal control cells did not reach statistical significance. In contrast, PA-derived ASM production was similar among PLIN5-transgenic and β-Gal control cells (Fig. 6D, middle). Overexpression of target proteins during the assay was verified via Western blot analysis (Fig. 6D, right). In summary, these data suggest that LDMC per se has a very moderate if any impact on mitochondrial FA oxidation, whereas overexpression of PLIN5 or PLIN5(Δ461–463) substantially but comparably interfered with TG catabolism and FA esterification into TG.

### LDMC promotes molecular interactions between PLIN5 and protein complexes involved in mitochondrial respiration and dynamics

**Fig. 7:**
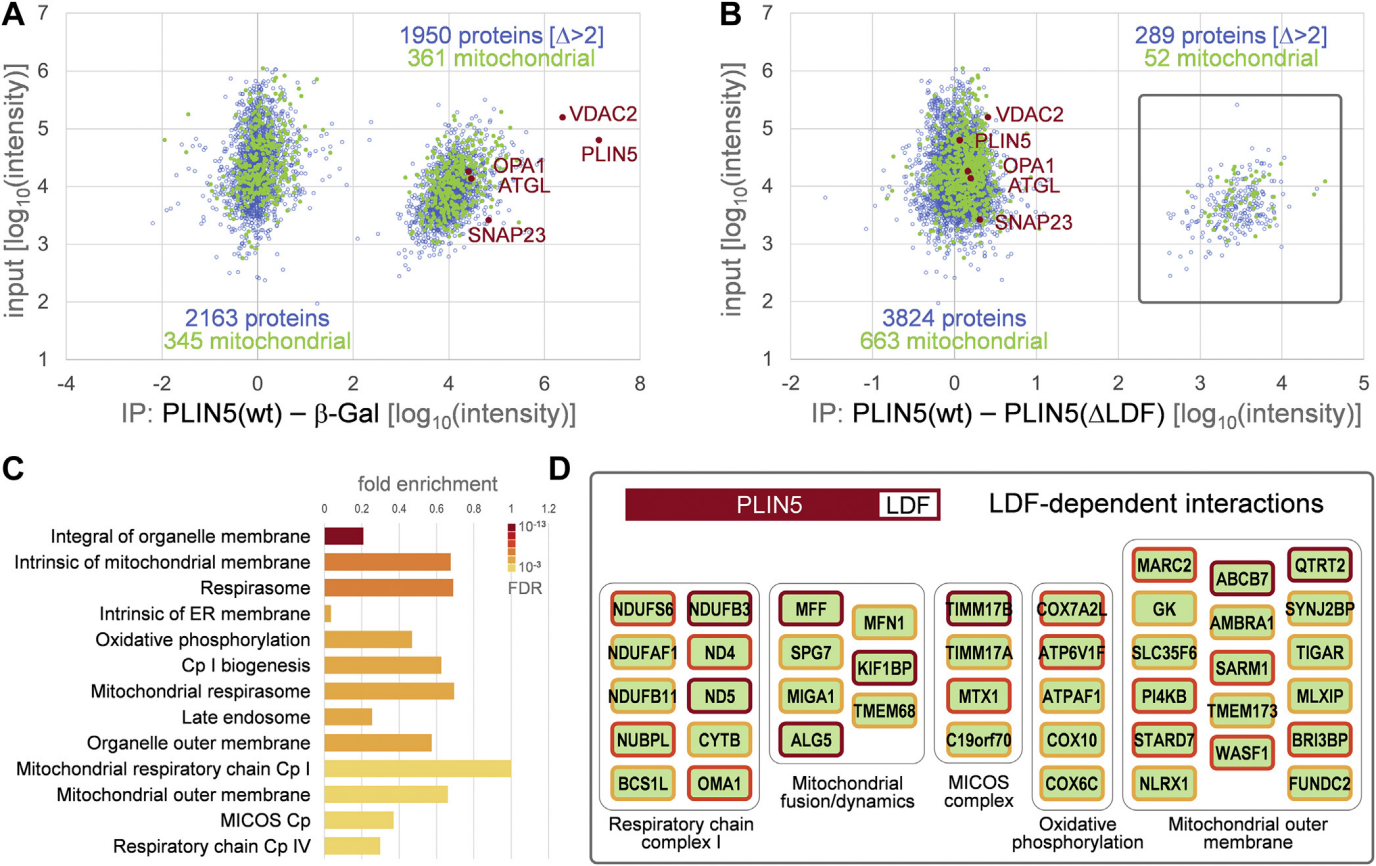
PLIN5 interacts with mitochondrial protein complexes involved in oxidative phosphorylation and mitochondrial dynamics. A: Scatterplot showing the relative intensity difference of proteins copurified with FLAG-PLIN5 and FLAG-β-Gal against the input intensities determined from the cell lysates. Every data point represents the average of two or three label-free quantifications from three biological replica experiments. Blue open circles: proteins. Green closed circles: GO annotated mitochondrial proteins. PLIN5, VDAC2, OPA1, ATGL, and SNAP23 are indicated as red closed circles. B: As in (A), label-free quantitative comparison of FLAG-PLIN5 versus FLAG-PLIN5(Δ461–463) coimmunoprecipitation experiments. About 289 proteins that copurify in two or more biological replica experiments with FLAG-PLIN5 but are not found with FLAG-PLIN5(Δ461–463) lacking the C-terminal Leu-Asp-Phe (LDF) sequence are boxed. C: GO term enrichment of proteins ranked by FLAG-PLIN5—FLAG-PLIN5(Δ461–463) intensity difference. Fold enrichment is displayed for top significant component categories. False discovery rate (FDR) is color coded. Cp = complex. GO: 0031301, integral component of organelle membrane; GO: 0098573, intrinsic component of mitochondrial membrane; CL: 22328, GO: 0070469, respirasome; GO: 0031227, intrinsic component of endoplasmic reticulum membrane; CL: 22327, GO: 0006119, oxidative phosphorylation; CL: 22331, GO: 0032981, complex I biogenesis; GO: 0005746, mitochondrial respirasome; GO: 0005770, late endosome; GO: 0031968, organelle outer membrane; GO: 0005747, mitochondrial respiratory chain complex I; GO: 0005741, mitochondrial outer membrane; CL: 22642, GO: 006617, mitochondrial protein import, and MICOS complex; CL: 22641, GO: 0008535, mitochondrial protein import, and respiratory chain complex IV assembly. D: Selected PLIN5 LDF-dependent interaction partners: Protein subunits of enriched complexes detected in FLAG-PLIN5(wt) but not FLAG-PLIN5(Δ461–463) co-IP experiments. Border colors: red, found in three, orange found in two, and yellow found in one of three replica experiments. MICOS, mitochondrial contact site and cristae organizing system; SNAP23, synaptosomal-associated protein 23; VDAC2, voltage-dependent anion-selective channel protein 2.

Although our experiments revealed only a minor reduction of β-oxidation rates upon disruption of PLIN5-mediated LDMC, decreased substrate oxidation may be indicative of damage to the electron transport chain (ETC) and impaired mitochondrial capacity (35). Furthermore, it is known that a 30–50% reduction of ETC enzyme activities is required to lower the rate of oxidative phosphorylation (OXPHOS) because of an excess of enzyme activities of ETC complexes relative to OXPHOS (35). Thus, we reasoned that disruption of LDMC may perturb mitochondrial function despite retained β-oxidation capacity. This prompted us to further explore the impact of LDMC on molecular interactions between PLIN5 and protein complexes involved in mitochondrial energy production and dynamics. Employing AC16 cardiomyocytes stably expressing FLAG-tagged PLIN5(Δ461–463), we investigated molecular interaction changes through IP-purification proteomics in comparison to PLIN5(wt)-expressing and β-Gal-expressing cells. Therefore, we isolated the FLAG-tagged proteins through IP in triplicates and identified copurifying protein complexes using parallel accumulation—serial fragmentation combined with data-independent acquisition MS (Fig. 7). Data are available via ProteomeXchange with identifier PXD028541. More than 5,100 proteins were identified in at least one IP experiment. Relative quantification of 4,113 proteins that were found in at least two biological replicas in the PLIN5(wt) IP revealed a large fraction of proteins copurifying with PLIN5(wt), which was not detectable in the β-Gal-control samples (Fig. 7A). Among the 1,950 proteins found specifically with PLIN5(wt), solely 361 proteins were annotated as components of mitochondria. In agreement with the literature, we recall the well-known interaction of PLIN5 with ATGL (8) and also copurified the mitochondrial proteins optic atrophy 1 and voltage-dependent anion-selective channel protein 2, as well as the SNARE (soluble *N*-ethylmaleimide-sensitive fusion protein attachment protein receptor) protein synaptosomal-associated protein 23 implicated in LDMC formation (29). The comparison between PLIN5(wt) and PLIN5(Δ461–463) IPs shows that the interactions with these four proteins are not significantly altered, confirming that deletion of the C-terminal Leu-Asp-Phe sequence of PLIN5 does not perturb protein interaction with ATGL (Fig. 7B). Interestingly, we found 289 proteins copurifying specifically with wild-type PLIN5 but not PLIN5(Δ461–463). The identified candidates included a large fraction of 52 mitochondrial proteins, indicating that molecular interactions between PLIN5 and these proteins were lost concomitantly with disruption of LDMC. Gene Ontology term and protein complex enrichment analyses revealed functionally consistent sets of proteins localized in the (outer) mitochondrial membrane, hinting toward complexes with mitochondrial respiratory activities (Fig. 7C). Moreover, binding of several NADH-ubiquinone oxidoreductase subunits of the mitochondrial respiratory complex I, as well as of proteins implicated in mitochondrial dynamics and cristae organization (including members of the mitochondrial contact site and cristae organizing system complex), was lost upon truncation of the last three C-terminal amino acids of PLIN5 (Fig. 7D). These findings suggest that PLIN5 may impact mitochondrial respiration beyond regulation of lipolysis via the interaction with mitochondrial proteins involved in mitochondrial respiration, cristae organization, and dynamics.

### PLIN5-mediated LDMC significantly augments the mitochondrial respiratory capacity and metabolic flexibility of AC16 cardiomyocytes

**Fig. 8:**
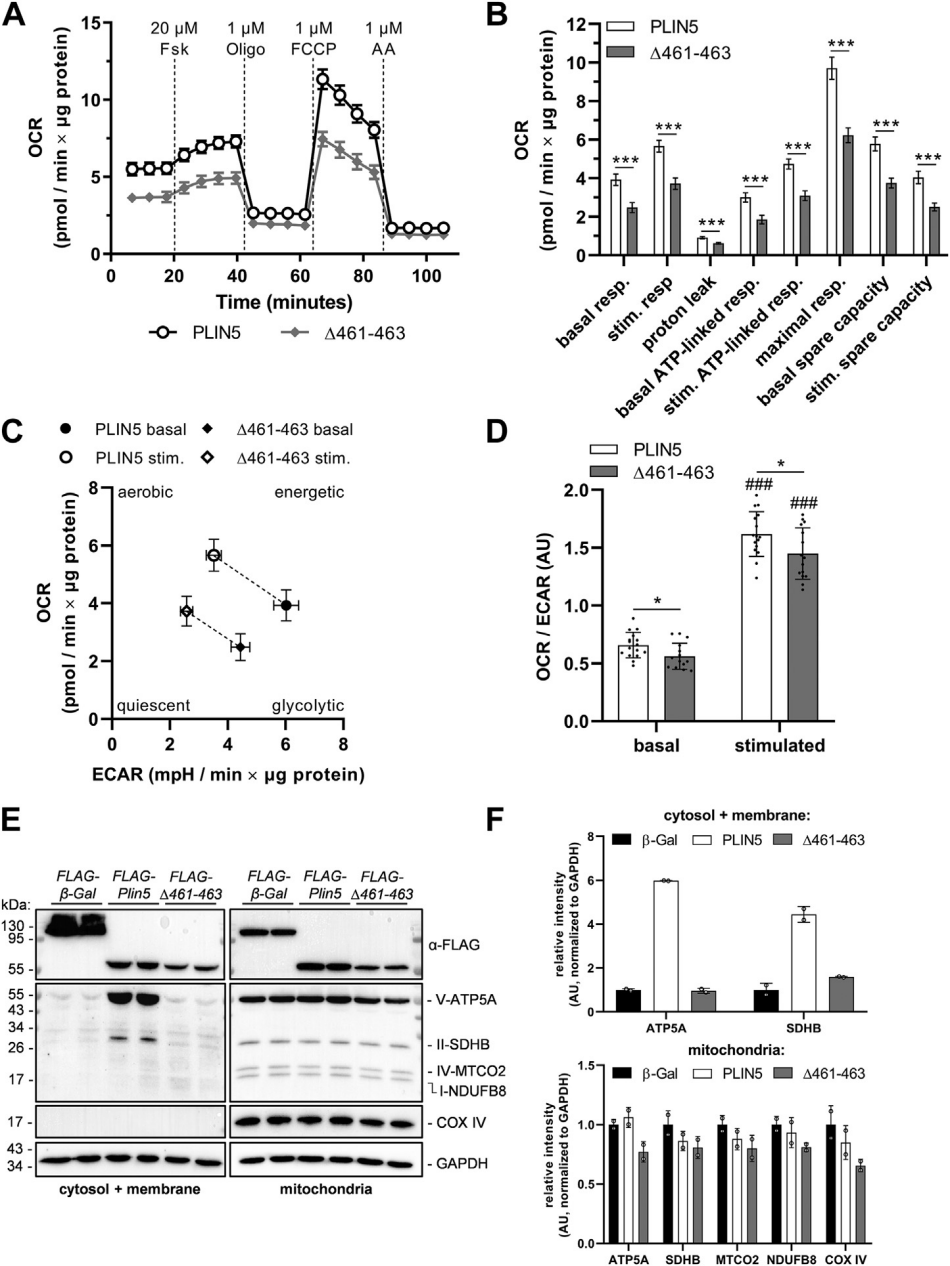
PLIN5-mediated LDMC augments the mitochondrial respiratory capacity and metabolic flexibility of lipid-challenged cardiomyocytes compared with cells overexpressing PLIN5(Δ461–463). AC16 cells stably overexpressing PLIN5 or PLIN5(Δ461–463) were cultured in the presence of 0.4 mM OA-BSA for 24 h, followed by respirometry analysis using a Seahorse XFe96 analyzer. Assay medium consisted of DMEM (catalog no.: D5030; Gibco) containing 5 mM glucose and 2 mM GlutaMAX, adjusted to pH 7.4 prior to measurement. A: OCR profiles upon sequential injection of forskolin (Fsk), oligomycin A (Oligo), carbonyl cyanide 4-(trifluoromethoxy)phenylhydrazone (FCCP), and antimycin A (AA), as indicated (n = 15–16). B: As measured in (A), quantification of basal, forskolin-stimulated (stim.), and maximal respiration (resp.), mitochondrial proton leak, as well as ATP-linked respiration and mitochondrial spare capacity under basal or stimulated conditions. C, D: Metabolic shift upon β-adrenergic stimulation was (C) visualized by plotting OCR against extracellular acidification rate (ECAR) and (D) quantified by calculating the OCR to ECAR ratio. E: Stable AC16 cells were treated with 0.4 mM OA-BSA for 24 h, followed by subcellular fractionation. Expression levels of ETC proteins, as indicated, in cytosol/membrane fractions or mitochondria-enriched fractions were determined by immunoblot analyses using an anti-total-OXPHOS-human antibody cocktail and an anti-COX IV antibody. GAPDH was probed as a marker for cytosolic crosscontamination and as loading control. F: Quantification of immunoblot signals (E) normalized to GAPDH. Data are presented as mean ± 95% CI (A, B) or mean ± SD (C, D). Statistical significance was determined by unpaired Student’s *t*-test (ns = not significant, ∗*P* < 0.05, ∗∗∗*P* < 0.001; ###*P* < 0.001 vs. corresponding basal conditions). AU, arbitrary unit.

Given the perturbed interaction of PLIN5(Δ461–463) with respiratory complex proteins, we next examined the impact of disrupted LDMC on mitochondrial respiratory capacity via Seahorse XF analysis in cells incubated in complete medium or in cells subjected to 24 h of loading with OA prior to Seahorse analysis. In line with our proteomics data, AC16 cells stably overexpressing PLIN5 exhibited significantly increased OCR, ATP-linked respiration, and mitochondrial spare capacity under both basal and forskolin-stimulated conditions relative to cells expressing PLIN5(Δ461–463) (Fig. 8 and supplemental Fig. S8). Notably, the reduction in OCR levels was even more pronounced in PLIN5(Δ461–463) transgenic cells compared with PLIN5 transgenic cells upon preloading with OA (Fig. 8A, B compared with supplemental Fig. S8A, B), which may indicate that stimulation of lipolysis (forskolin treatment) in the absence of LDMC adversely impacts mitochondrial function and respiratory capacity. The mitochondrial capacity of PLIN5(Δ461–463)-expressing cells was also significantly reduced compared with β-Gal control cells under the tested conditions (data not shown). Furthermore, LDMC significantly enhanced the metabolic shift from glycolysis toward mitochondrial respiration upon forskolin stimulation (supplemental Fig. S8C, D). Again, this effect was even more pronounced in OA-challenged cells (Fig. 8C, D). In accordance with these results, subcellular fractionation of OA-treated AC16 cells revealed decreased expression levels of several ETC complex proteins in mitochondria-enriched fractions from PLIN5(Δ461–463)-expressing cells relative to PLIN5-overexpressing or β-Gal-overexpressing cells (Fig. 8E, F). This was true despite similar mitochondrial DNA content upon disruption of LDMC (supplemental Fig. S9). Interestingly, we also detected strong signals of ATP synthase subunit alpha (ATP5A) as well as succinate dehydrogenase specifically in the cytosol/membrane fraction of PLIN5-overexpressing cells (Fig. 8E, F), suggesting that a subpopulation of mitochondria remained attached to LDs during the subcellular fractionation because of the tight coupling of LDs and mitochondria via PLIN5. These data indicate overall higher protein levels of ATP synthase and succinate dehydrogenase upon interaction of mitochondria with PLIN5, which is in line with improved mitochondrial respiratory function.

PLIN5 has been demonstrated to act as a nuclear transcriptional coactivator involved in the regulation of PPAR γ coactivator 1 alpha (PGC-1α) target gene expression and thereby interfering with mitochondrial respiration and function (17, 18, 19). It is conceivable that disrupted mitochondrial interaction of PLIN5(Δ461–463) also impacts nuclear shuttling and consequently coregulation of PGC-1α target gene expression. To address this assumption, we investigated the nuclear abundance of PLIN5 versus PLIN5(Δ461–463) in transgenic AC16 cells (supplemental Fig. S10). Western blot analyses revealed similar nuclear abundance of PLIN5 compared with PLIN5(Δ461–463) during basal and forskolin-stimulated conditions (supplemental Fig. S10A, B) suggesting that nuclear shuttling of PLIN5 is not affected by disrupted LDMC. Moreover, mRNA expression levels of PDK4 (supplemental Fig. S10C), an established PGC-1α target gene (36), are comparable among AC16 cells overexpressing PLIN5 or PLIN5(Δ461–463), respectively. Together, these findings suggested that the observed differences in mitochondrial respiratory function upon overexpression of PLIN5 compared with PLIN5(Δ461–463) are independent of PLIN5 acting as a nuclear transcriptional coregulator.

## Discussion

Since its identification in 2006 (2, 3, 4), PLIN5 has emerged as an essential regulator of lipolysis in oxidative tissues, where PLIN5 targets lipolytic proteins to the LD surface and coordinates their interactions (5, 6, 7, 8, 9). Besides, PLIN5 has the unique characteristic to interact with mitochondria likely via the last 20 amino acids, thereby tightly anchoring mitochondria to LDs (20), which we termed LDMC. However, the role of LDMC in mitochondrial energy catabolism and β-oxidation is controversially discussed and awaits further clarification.

To study the specific role of PLIN5-mediated LDMC in cellular energy catabolism, it is mandatory to overexpress mutant PLIN5 that does not impact the physiological regulation of lipolysis and consequently the dynamic interaction with ATGL and CGI-58 (and hormone-sensitive lipase) but abrogates LDMC. Intracellular TG levels are mainly determined by continuous cycles of TG synthesis, TG hydrolysis, and FA re-esterification into TG among other lipids. We demonstrate that stable overexpression of PLIN5(Δ461–463) solely lacking the last three C-terminal amino acids markedly disrupted LDMC, whilst not significantly altering intracellular TG homeostasis compared with cells overexpressing full-length PLIN5. This was observed in cells cultivated in complete medium as well as in cells loaded with OA followed by serum starvation and forskolin-induced β-adrenergic stimulation. These findings implicated that lipolysis is not divergently affected by overexpression of PLIN5(Δ461–463) compared with PLIN5. In line with this assumption, incubation with triacsin C and consequently inhibition of FA esterification thereby promoting FA release was only marginally reduced in PLIN5(Δ461–463)-overexpressing cells compared with PLIN5-overexpressing cells. However, these findings contradict a previous study showing that PLIN5-mediated LDMC promotes TG synthesis compared with abrogated LDMC (23). In this study, PLIN5(Δ399–463) was overexpressed in brown adipocytes to examine the role of LDMC in lipid turnover. However, co-IP assays revealed that a similar PLIN5(Δ424–463) mutant markedly reduced the interaction with ATGL and thus may interfere with lipolysis and TG homeostasis. Diminished interaction of PLIN5(Δ424–463) and likely PLIN5(Δ399–463) with ATGL is in accordance with the study by Granneman *et al.* (8) demonstrating that the PLIN5(Δ400–463) mutant interacts neither with ATGL nor with CGI-58. Moreover, IP assays applying various PLIN5 truncation variants revealed that the region spanning amino acids 424–443 of PLIN5 is critical for efficient interaction with ATGL.

Together, these findings also validated PLIN5(Δ461–463)-overexpressing cells as a model to study the impact of abolished LDMC on energy catabolism compared with PLIN5-mediated LDMC. We hypothesized that LDMC and the close proximity of LDs and mitochondria interferes with FA oxidation. To address this assumption, we examined FA oxidation in three different cell lines (COS-7, AML-12, and AC16 cells) and during different experimental setups including PA incubation or preincubation with a mixture of OA and PA to promote PA incorporation into cellular TG followed by stimulation of lipolysis. However, overexpression of both PLIN5 or PLIN5(Δ461–463) moderately reduced β-oxidation (^14^CO_2_ release) to a similar degree compared with LacZ control. Nonetheless, we measured marginally but significantly reduced ^14^CO_2_ release in AML-12 hepatocytes overexpressing PLIN5(Δ461–463) compared with PLIN5, whereas solely overexpression of PLIN5(Δ461–463) caused a moderate but significant decrease in β-oxidation of forskolin-stimulated AC16 cardiac cells when compared with LacZ control. These findings suggested that LDMC has a very moderate if any impact on mitochondrial FA oxidation rates and that overexpression of PLIN5 or PLIN5(Δ461–463) reduces mitochondrial β-oxidation mainly via deceleration of TG hydrolysis. This is also in line with the outcome of the pulse-chase experiments and a similar impact on TG homeostasis, which is determined by TG synthesis, lipolysis, FA re-esterification, and oxidation depending on the cellular energy requirements. In accordance with the assumption that LDMC does not advance mitochondrial FA oxidation, Nguyen *et al.* (37) demonstrated that LDs are not required for FA delivery to mitochondria but instead protect mitochondria from acylcarnitine accumulation and lipotoxic dysfunction. Particularly interesting, OCRs, as a measure of mitochondrial ETC activity, were significantly reduced in PLIN5(Δ461–463)-overexpressing AC16 cardiomyocytes presented with disrupted LDMC compared with PLIN5-induced LDMC. Notably, OCRs further decreased in PLIN5(Δ461–463) cells upon preincubation with OA, which may indicate increased mitochondrial stress upon a shift to more FA utilization as energy fuel. Moreover, OCR/extracellular acidification rate ratios, as a measure of oxidative versus glycolytic pathways for ATP production, were significantly increased in the basal and stimulated (forskolin) states in PLIN5 compared with PLIN5(Δ461–463)-overexpressing AC16 cells suggesting that mitochondrial energy catabolism and function are improved in cells with augmented LDMC compared with disrupted LDMC. Interestingly, PLIN5 has also been discovered as a transcriptional coregulator to positively regulate PGC-1α target gene expression (17, 18, 19) and thus may indirectly interfere with mitochondrial energy catabolism. The very similar nuclear abundance of PLIN5 compared with PLIN5(Δ461–463) indicates that changes in mitochondrial respiration upon PLIN5 compared with PLIN5(Δ461–463) overexpression are not apparently affected by changes in PGC-1α target gene expression.

Finally, what is the physiological role of PLIN5-mediated LDMC in cellular energy metabolism? From the numerous studies (more than 60) where PLIN5 was overexpressed or deleted/downregulated in cells or in mice, more than 25 studies directly or indirectly demonstrate that the deletion or downregulation of PLIN5 causes cellular stress and/or mitochondrial dysfunction linked to increased (and likely uncontrolled) FA oxidation, lipotoxicity, and/or insulin resistance (11, 12, 38, 39, 40, 41). Moreover, numerous studies demonstrate that overexpression of PLIN5 increases cellular TG levels, whereas no study exists showing the opposite. Nonetheless, a limited number of studies show that PLIN5 overexpression raises FA oxidation. However, effects were mostly very moderate (20, 25, 42) except for one study (4), and in some studies, FA oxidation was not measured but assumed from increased PPAR target gene expression. A (moderate) increase in FA oxidation upon overexpression of PLIN5 may also derive from protecting mitochondria from lipotoxic events upon incubation with PA. However, our study does not exclude the possibility that PLIN5 may locally and transiently enhance FA flux from LDs into mitochondria, thereby protecting a distinct population of mitochondria from a local rise of nonesterified FA levels and mitochondrial damage.

PLIN5 is highly expressed in oxidative tissues, which are temporarily exposed to high FA uptake and the transient deposition of FAs within LDs including cardiac and skeletal muscle and liver. Except for BAT, these tissues are not characterized by sustained TG storage. Increased levels of nonesterified FAs have been shown to cause cellular stress and mitochondrial dysfunction known as lipotoxicity (43). Augmented cellular FA uptake and stimulation of PPARα-target gene expression including PLIN5 expression (2, 4) triggers LD formation and LDMC (24, 28), which could act as signal to adapt mitochondria to increased FA oxidation and respiration. In line with this assumption, IP-purification proteomics revealed that PLIN5-mediated LDMC promoted significant interactions with several NADH-ubiquinone oxidoreductase proteins from respiratory complex I and other proteins involved in OXPHOS and mitochondrial dynamics, which were abrogated upon disrupted LDMC. Strikingly, we also observed PLIN5 interaction with several members of the mitochondrial contact site and cristae organizing system complex that plays a critical role in the formation and remodeling of mitochondrial cristae, which encompass the majority of OXPHOS complexes (44, 45). Particularly interesting, Varghese *et al.* (24) demonstrated that enhanced adipocyte lipolysis increases PLIN5 expression and LD expansion in the murine heart, which is paralleled by increased LDMC, accumulation of PLIN5 at the LD-mitochondria interface, and changes in mitochondrial cristae abundance and orientation. In line, a very recent study by the group of Perry Bickel linked BAT-specific PLIN5 overexpression to tighter packaging of mitochondrial cristae and increased uncoupled respiratory capacity in BAT, whereas PLIN5 deficiency reduced mitochondrial respiration and decreased cristae packing in brown adipocytes upon cold stress (46). To summarize, our study reveals an important role of PLIN5-mediated LDMC in mitochondrial respiratory function. The PLIN5-mediated brake on lipolysis may act as a lipid buffering system that sequesters FAs within TGs to protect cells from lipotoxic damage, and LDMC may adapt mitochondria to augmented cellular FA uptake and oxidation.

## Data availability

All data are contained within the article and in the supplemental data section and are available from the corresponding author upon reasonable request. MS proteomics data are available via ProteomeXchange (www.proteomexchange.org) with identifier PXD028541.

## Supplemental data

This article contains supplemental data.

## Conflict of interest

The authors declare that they have no conflicts of interest with the contents of this article.
