# Supplementary material for: Lipid droplet-mitochondria coupling via perilipin 5 augments respiratory capacity but is dispensable for FA oxidation
Source: J Lipid Res. 2022 Jan 21;63(3):100172. doi: 10.1016/j.jlr.2022.100172 (PMC8953689; doi:10.1016/j.jlr.2022.100172)
Supplement: Supplemental Figures S1–S10 and Table S1 [file mmc1.docx]

**SUPPLEMENTAL DATA:**

**Lipid droplet-mitochondria coupling via Perilipin 5 is dispensable for FA oxidation but augments respiratory capacity**

Benedikt Kien^1^, Stephanie Kolleritsch^1^, Natalia Kunowska^2^, Christoph Heier^1, 3, 4^, Gabriel Chalhoub^1^, Anna Tilp^1^, Heimo Wolinski^1, 4^, Ulrich Stelzl^2, 3, 4^, Guenter Haemmerle^1, 3, 4 *^

^1^Institute of Molecular Biosciences, University of Graz, 8010 Graz, Austria

^2^Institute of Pharmaceutical Sciences, Department of Pharmaceutical Chemistry, University of Graz, 8010 Graz, Austria

^3^BioTechMed-Graz, 8010 Graz, Austria

^4^Field of Excellence BioHealth - University of Graz, 8010 Graz, Austria

*Correspondence: Guenter Haemmerle, Institute of Molecular Biosciences, Heinrichstrasse 31/II, University of Graz, 8010 Graz, Austria. Email: [guenter.haemmerle@uni-graz.at](mailto:guenter.haemmerle@uni-graz.at)

**Supplemental Table 1. Primer pairs used for site-directed mutagenesis to generate C-terminally truncated PLIN5 variants.** Mismatch sequences introducing nucleotide substitutions in the *Plin5* CDS are highlighted in grey.

| primer name | primer sequence |
| --- | --- |
| (∆444-463)-F | 5’-TCCTGGGGGGTAAGAGGCTGAGC-3’ |
| (∆444-463)-R | 5’-TCTGCAGACTCAGCCTCCCAG-3’ |
| (∆454-463)-F | 5’-GGGGCAAGGCTAGCACACAATGAT-3’ |
| (∆454-463)-R | 5’-CTTGGGGGCTCAGCCTCT-3’ |
| (∆456-463)-F | 5’-AGGCAAGCACTAAATGATGCCAGAGCTGGACTTC-3’ |
| (∆456-463)-R | 5’-TGCCCCCTTGGGGGCTCA-3’ |
| (∆461-463)-F | 5’-GATGCCAGAGTAGGACTTCTGAATGG-3’ |
| (∆461-463)-R | 5’-ATTGTGTGCTTGCCTTGC-3’ |


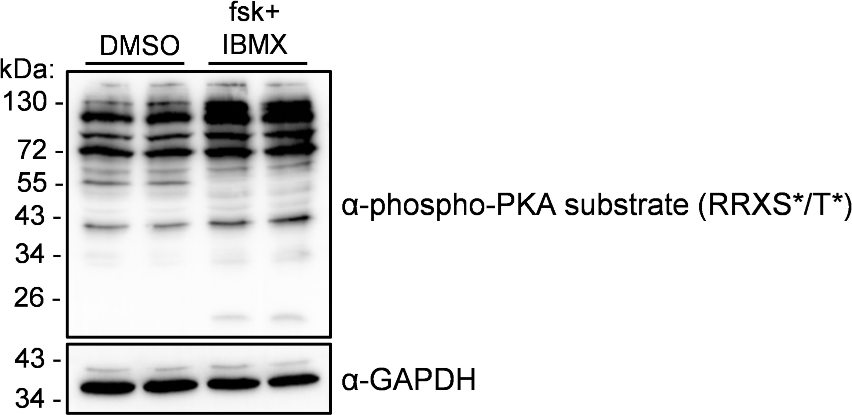


**Supplemental Fig. S1. Forskolin-treatment induces PKA kinase activity in AML12 hepatocytes.** AML12 cells were cultured in growth medium containing DMSO (vehicle) or 20 µM forskolin (fsk) together with 500 µM IBMX for two hours, to stimulate adenylate cyclase. Cells were lysed in buffer A containing phosphatase inhibitor (4906845001, Roche). Immunoblot analysis using an anti-phospho-PKA substrate antibody verified activation of PKA kinase activity upon forskolin treatment.

**
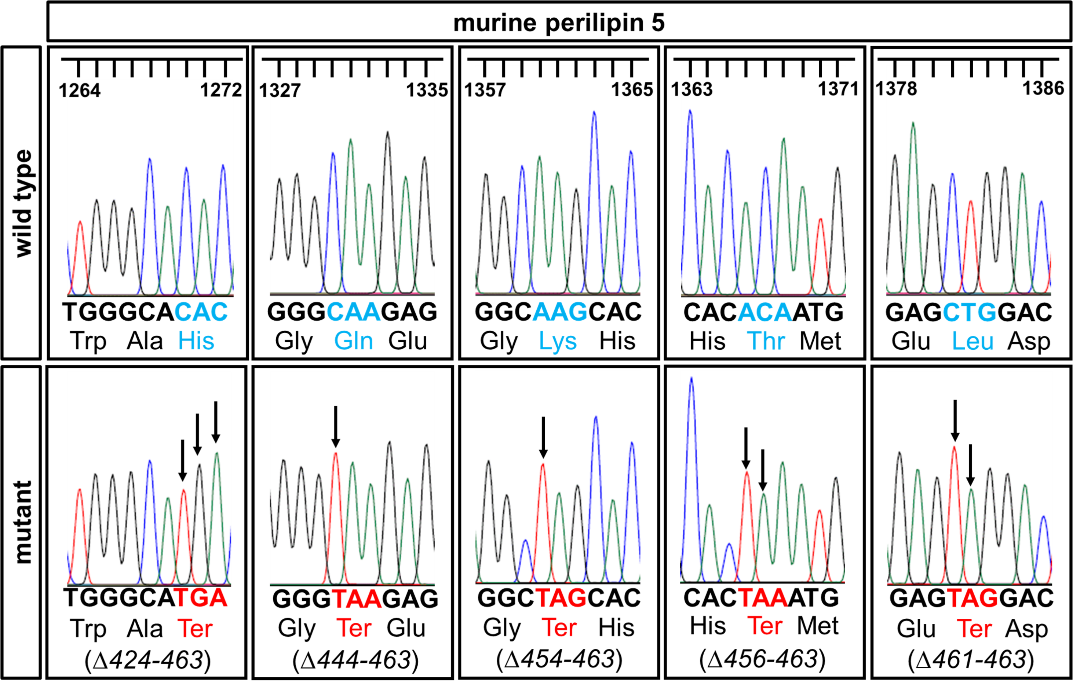
**

**Supplemental Fig. S2. DNA sequence analysis of vector constructs encoding C-terminally truncated PLIN5 variants.** Following site-directed mutagenesis, the generated expression vectors were validated by Sanger DNA sequencing (Microsynth Austria GmbH, Vienna, Austria). Chromatogram alignment confirmed insertion of stop codons in the *Plin5* coding sequence at the expected positions (arrows).


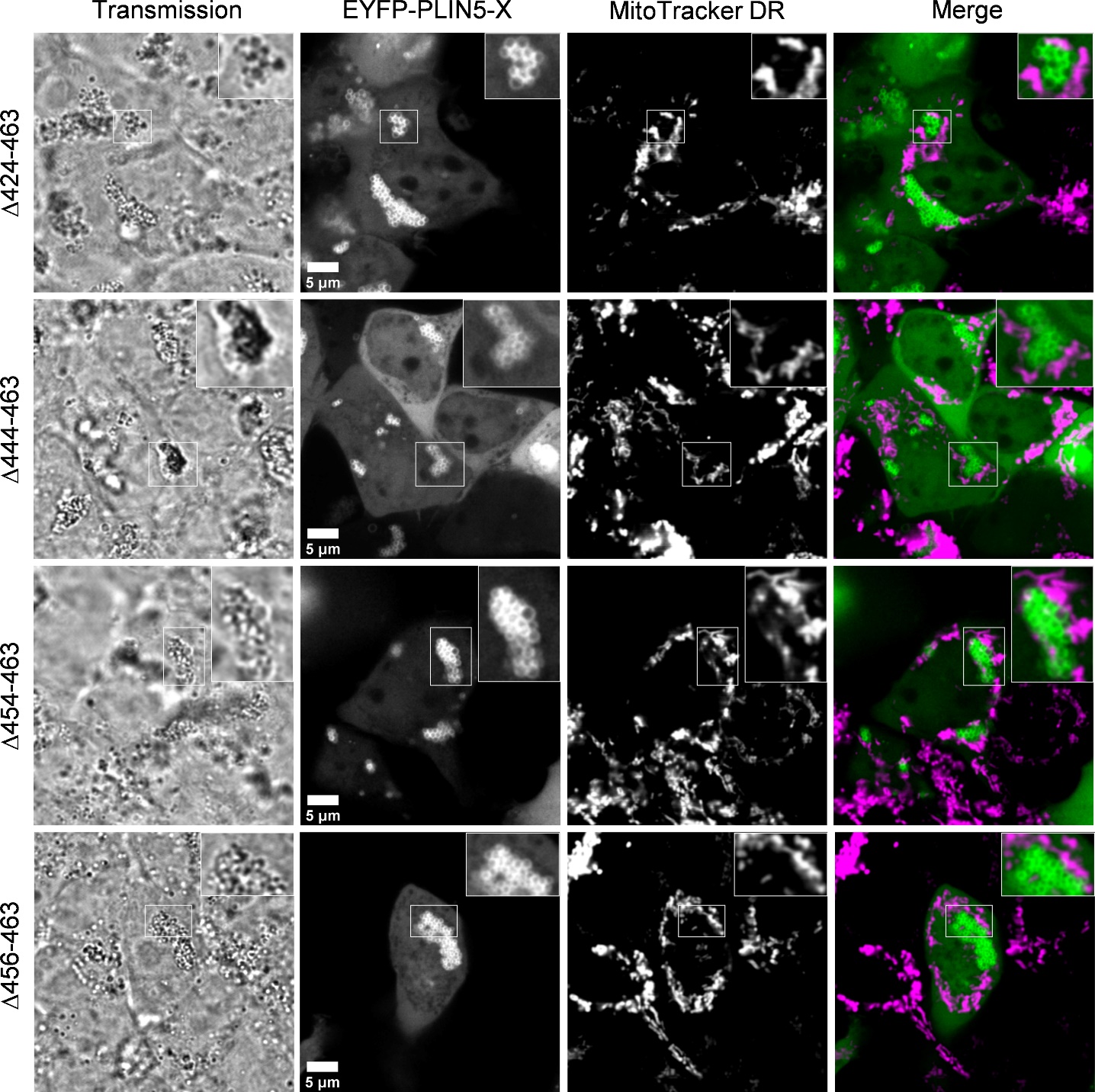


**Supplemental Fig. S3. Live cell imaging of HEK-293T cells transiently overexpressing C-terminally truncated PLIN5 variants.** HEK-293T cells were transfected with plasmid DNA encoding EYFP-tagged mutant PLIN5 variants as indicated. Cells were cultured in medium containing 0.4 mM OA-BSA overnight, to promote LD formation. Prior to confocal microscopy analysis, mitochondria were stained using MitoTracker DR. Neither of the tested PLIN5 mutants induced LDMC. Insets, 2× magnification.


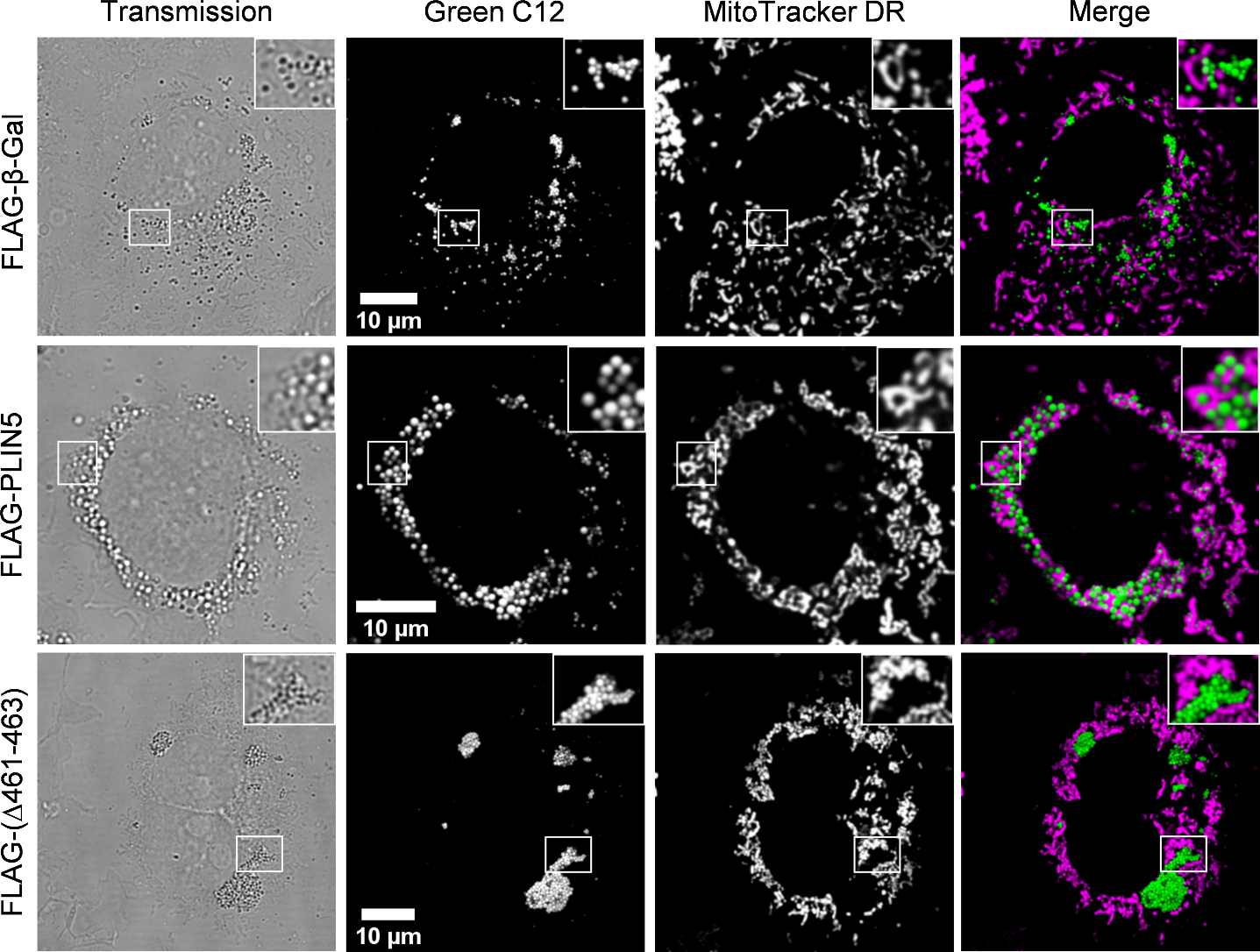


**Supplemental Fig. S4. PLIN5-mediated LDMC is abolished in COS7 cells upon overexpression of mutant PLIN5(∆461-463).** Transgenic COS-7 fibroblasts were incubated in medium containing 2 µM Green C12 overnight, followed by washing with PBS, one-hour incubation in complete medium, mitochondrial staining with MitoTracker DR and confocal live cell microscopy. Note the accumulation of perinuclear LD clusters in the PLIN5-expressing cells and the absence of LDMC in cells expressing the PLIN5(∆461-463) variant. Insets, 2× magnification.


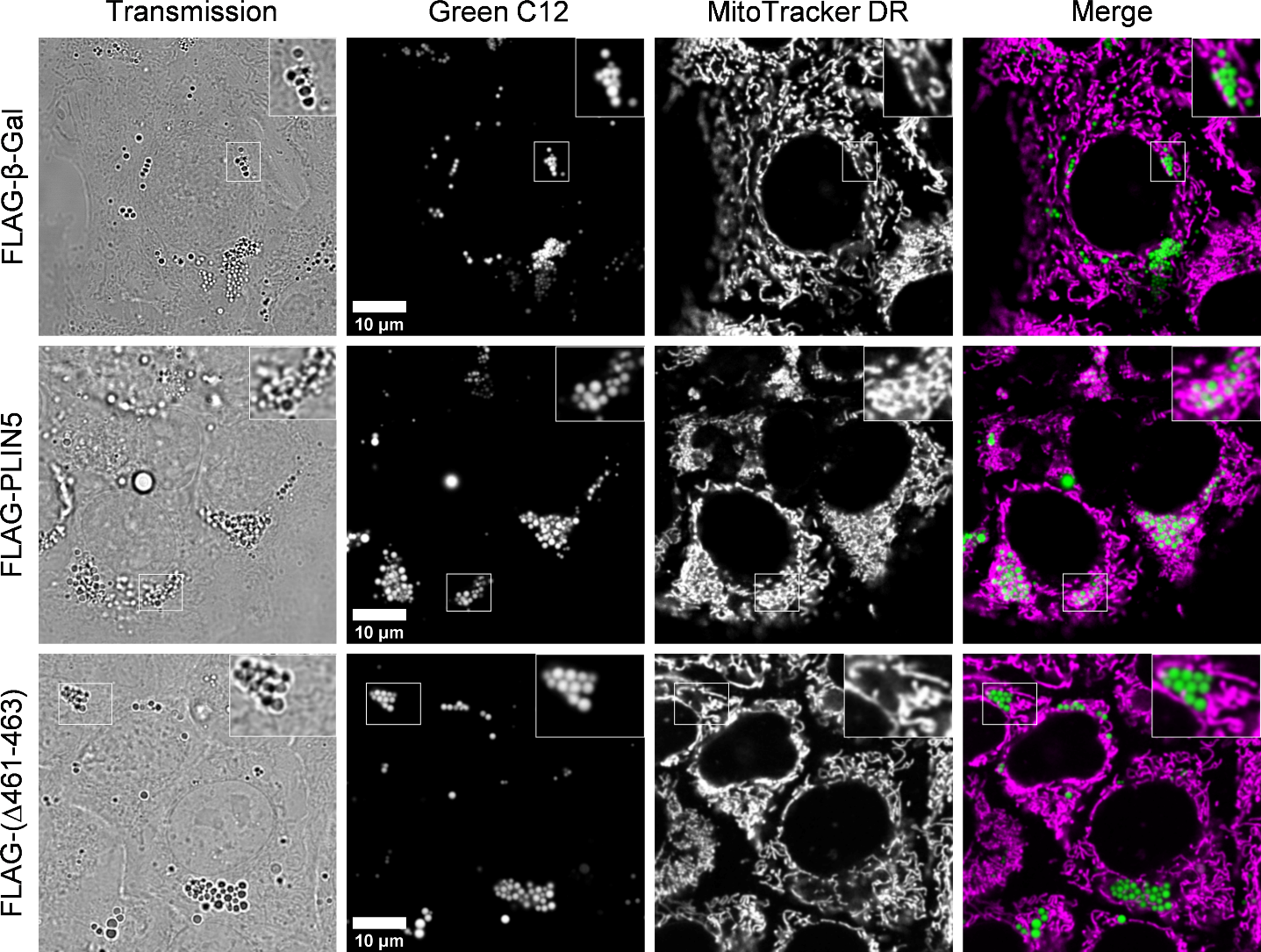


**Supplemental Fig. S5. PLIN5-mediated LDMC is disrupted in AML12 hepatocytes stably overexpressing the PLIN5(∆461-463) truncation variant.** Lentivirus-transduced AML12 cells expressing FLAG-tagged β-Gal, PLIN5 or PLIN5(∆461-463) were treated as described in supplemental Fig. S4 and subjected to confocal live cell imaging. Insets, 2× magnification.


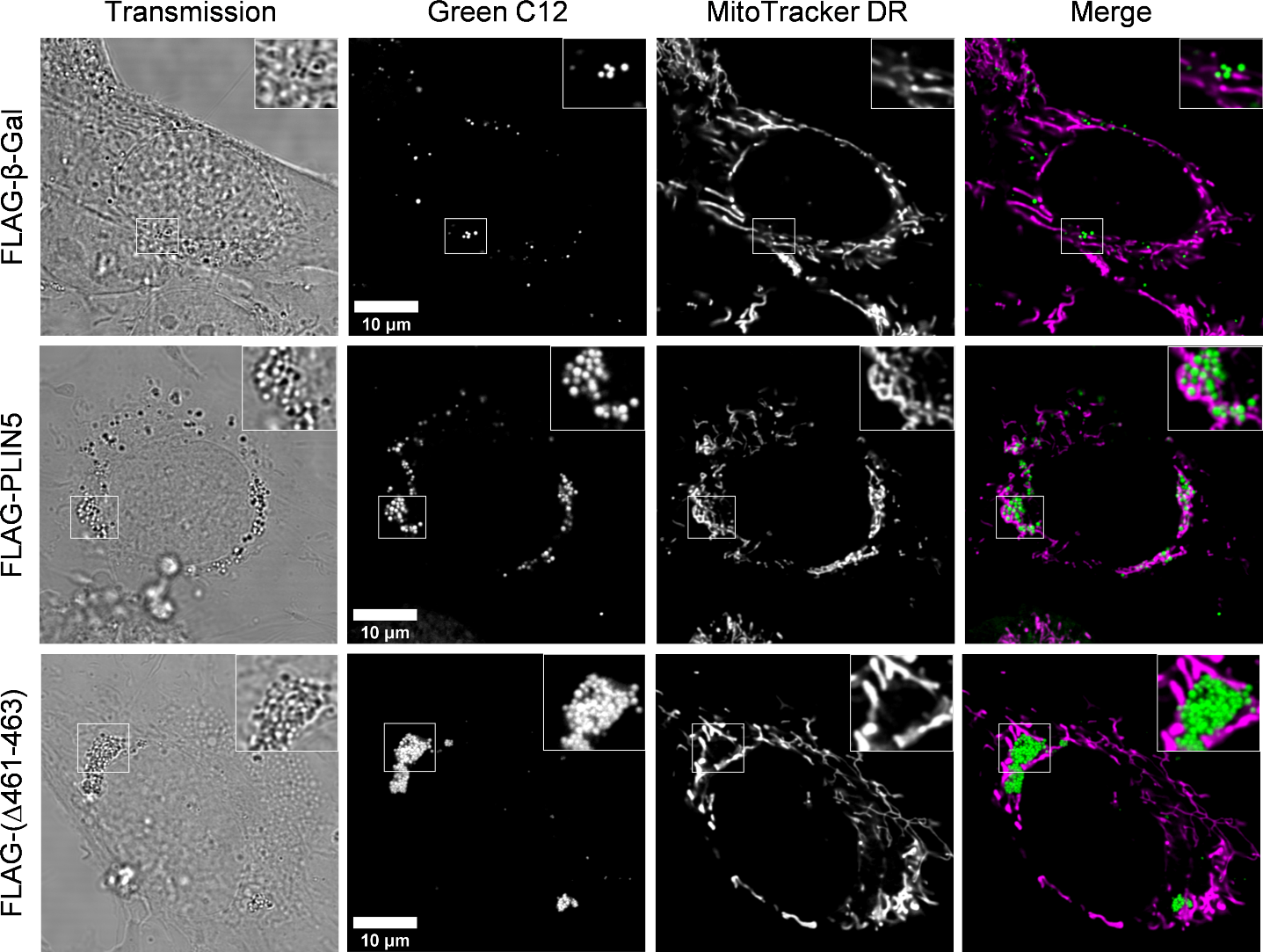


**Supplemental Fig. S6. PLIN5-mediated LDMC is abolished in AC16 cardiomyocytes stably overexpressing PLIN5(∆461-463).** AC16 cells stably overexpressing FLAG-tagged β-Gal, PLIN5 or PLIN5(∆461-463) were treated with Green C12 as described in supplemental Fig. S4. Following mitochondrial staining with MitoTracker DR, cells were examined by live cell microscopy. Insets, 2× magnification.


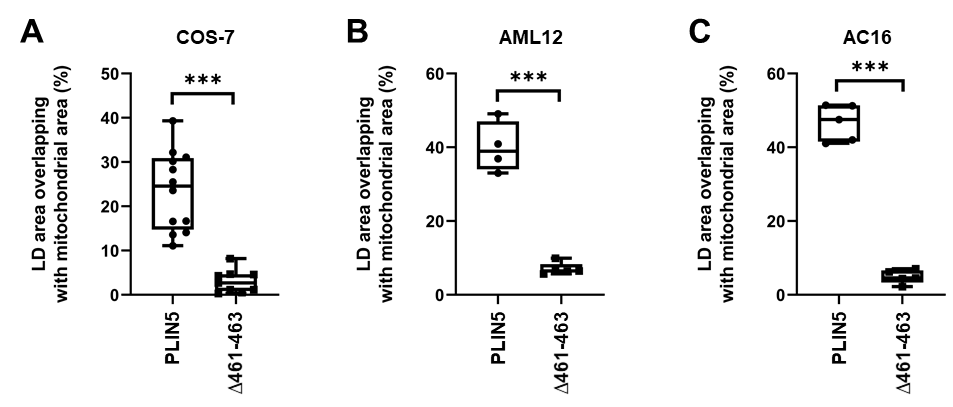


**Supplemental Fig. S7. Image-based quantification of the recruitment of mitochondria to LDs in COS-7, AML12 and AC16 cells stably expressing wild type or mutant PLIN5.** > 20 cells were analyzed for each cell type. Data are shown as mean ± SD. Statistical significance was determined by unpaired Student’s t-test (***p < 0.001).


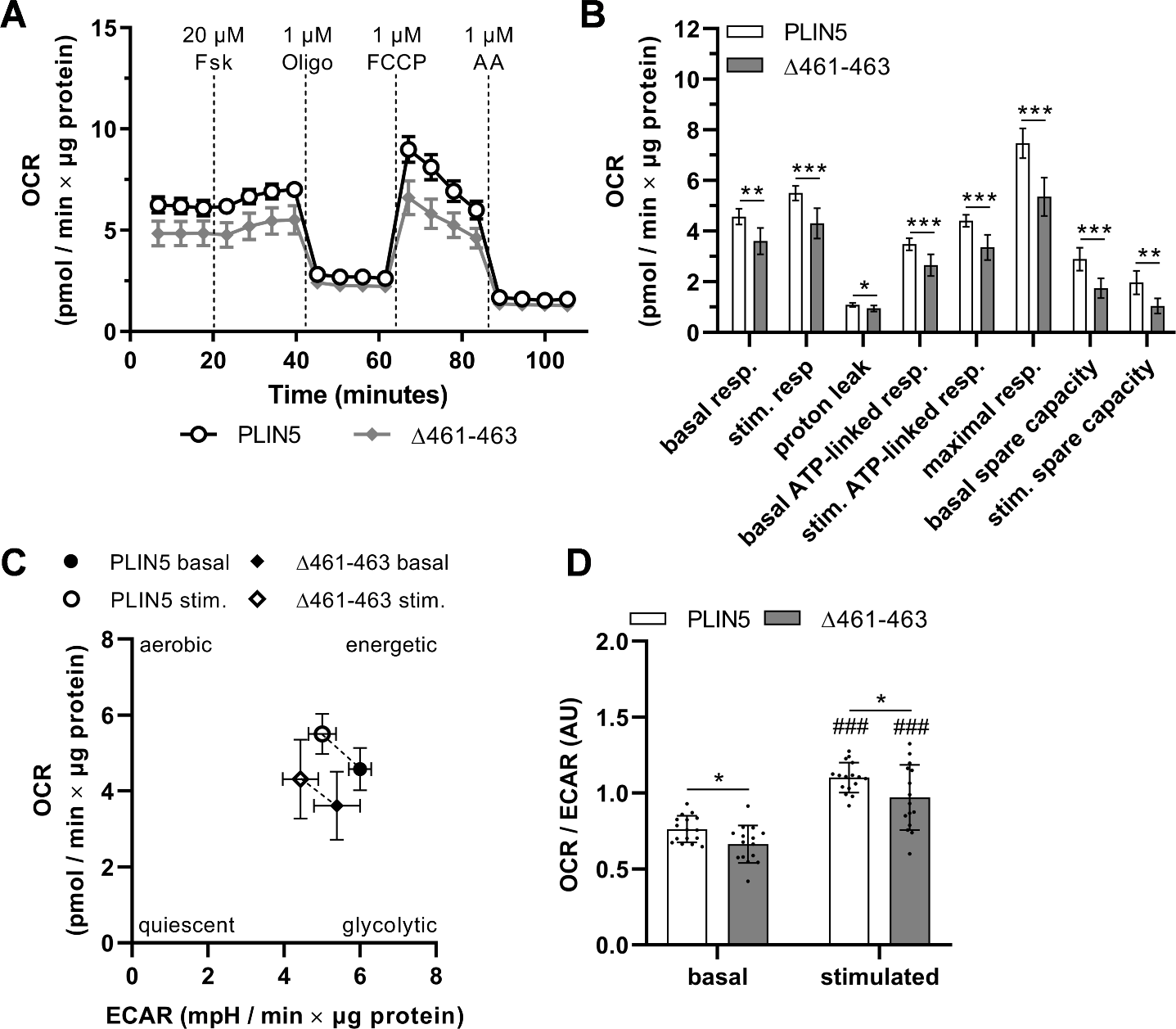


**Supplemental Fig. S8. PLIN5-mediated LDMC augments the mitochondrial respiratory capacity and metabolic flexibility of cardiomyocytes compared to cells overexpressing PLIN5(∆461-463).** AC16 cells stably expressing wild type or mutant PLIN5 were cultured in complete medium for 24 hours, followed by Seahorse XFe96 extracellular flux analysis. Assay medium consisted of DMEM (D5030, Gibco) supplemented with 5 mM glucose and 2 mM GlutaMAX, adjusted to pH 7.4 prior to measurement. **A:** Oxygen consumption rate (OCR) profile upon sequential injection of forskolin (Fsk), oligomycin A (Oligo), carbonyl cyanide 4-(trifluoromethoxy)phenylhydrazone (FCCP) and antimycin A (AA), as indicated (*n* = 15-16). **B:** Metabolic parameters under both, basal and forskolin-stimulated (stim.) conditions were quantified based on the data measured in (A). **C, D:** Metabolic shift upon forskolin-stimulation was analyzed by (C) plotting OCR against ECAR as well as by (D) calculation of the OCR to ECAR ratio. Data are presented as mean ± 95% CI (A, B) or mean ± SD (C, D). Statistical significance was determined by unpaired Student’s *t*-test (ns = not significant, **p* < 0.05, ***p* < 0.01, ****p* < 0.001; ###*p* < 0.001 vs. corresponding basal conditions). Resp., respiration; AU, arbitrary unit.


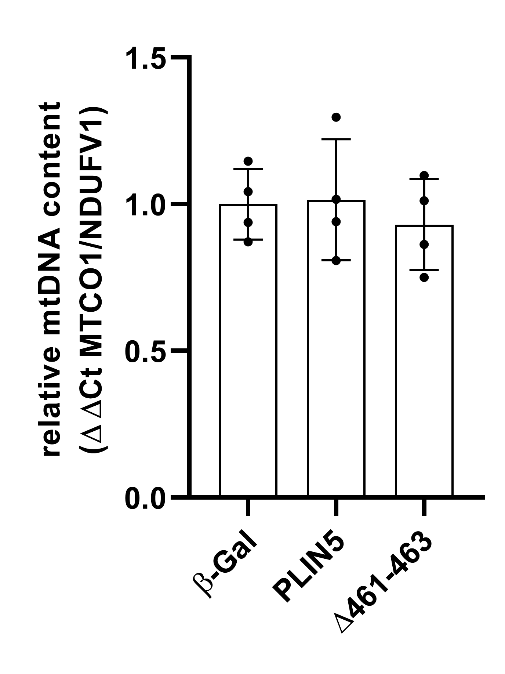


**Supplemental Fig. S9. Disruption of PLIN5-mediated LDMC has no impact on mitochondrial DNA content.** Total DNA from AC16 cardiomyocytes stably expressing FLAG-tagged recombinant proteins was isolated and analyzed by qPCR. Relative mtDNA content was calculated from copy numbers of the mitochondrial encoded *MTCO1*/nuclear DNA-encoded *NDUFV1* gene ratios. Data are presented as mean ± SD (*n* = 4). Statistical significance was determined by unpaired Student’s *t*-test.


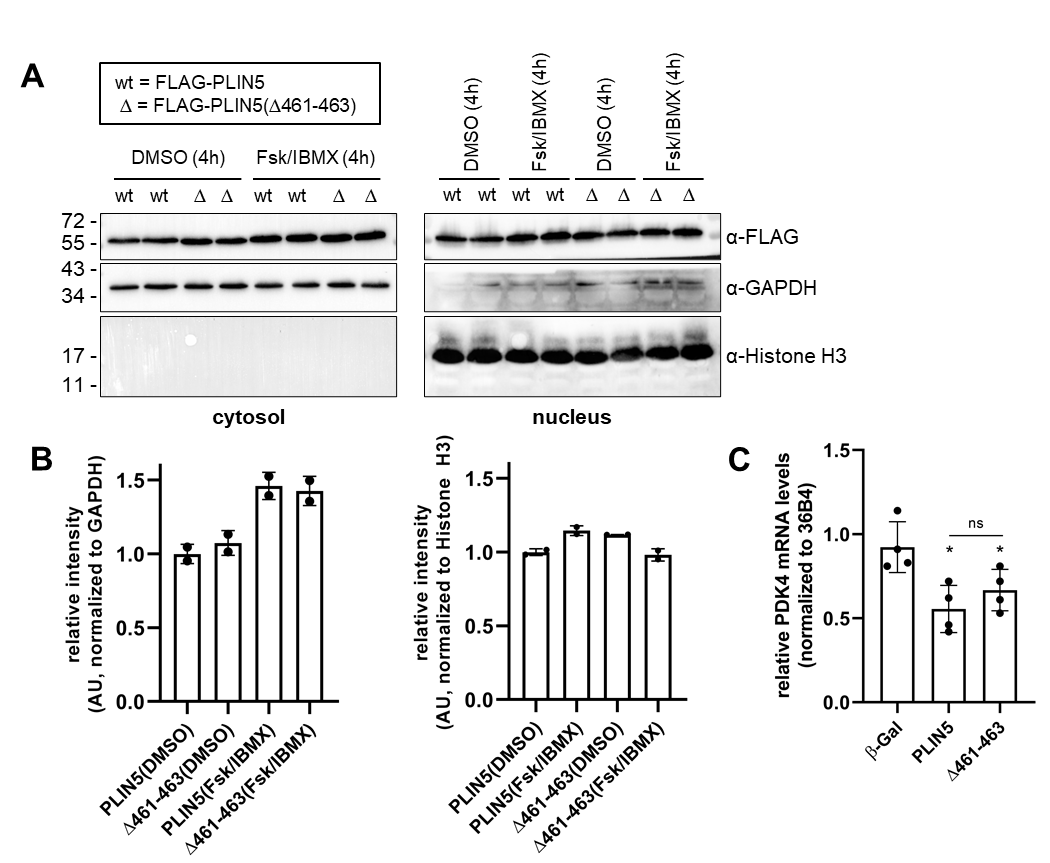


**Supplemental Fig. S10. Nuclear shuttling of PLIN5 is not impaired upon truncation of its last three C-terminal amino acids. A:** HEK-293T cells were transfected with plasmid DNA encoding FLAG-tagged wild type or mutant PLIN5. Subsequently, the cells were cultured for 4 hours in medium containing DMSO (vehicle) or 20 µM forskolin (fsk) together with 500 µM IBMX to stimulate PKA kinase activity. Following subcellular fractionation via the REAP method, the cytosolic and nuclear levels of the PLIN5 variants were determined by immunoblot analyses using an anti-FLAG antibody. Histone H3 and GAPDH were probed as nuclear and cytosolic markers, respectively. **B:** Quantification of immunoblot signals (A) normalized to GAPDH (cytosol) or Histone H3 (nucleus). **C:** *PDK4* mRNA levels relative to *36B4* reference gene in transgenic AC16 cells, as determined by qPCR analysis. Data are presented as mean ± SD (n = 4). Statistical significance was determined by unpaired Student’s t-test (ns = not significant, *p < 0.05).

**Supplemental materials and methods**

**Plasmids, cloning of recombinant proteins and site-directed mutagenesis**

cDNAs encoding murine *Plin5* (NM_025874.3), *Atgl* (NM_001163689.1) or *Cgi-58* (NM_026179.2) were cloned into the pcDNA4/HisMaxC expression vector (Invitrogen Life Technologies), as previously described (1–3). A pcDNA4/HisMax vector encoding *E. coli* β-galactosidase (β-Gal) was provided by the manufacturer (Invitrogen Life Technologies). Insert-DNA fragments with unique restriction site overhangs were amplified by PCR from respective cDNAs using Q5 high-fidelity DNA polymerase (M0491, NEB) and purified by agarose gel electrophoresis. Subsequently, the DNA fragments were co-digested with adequate restriction enzymes, followed by ligation into expression vectors with compatible restriction sites using T4 DNA ligase (M0202, NEB). FLAG-β-Gal-encoding cDNA was amplified from pcDNA4/HisMax-lacZ using the primers 5’-ATA CTC GAG CCA TGG ATT ACA AGG ACG ACG ATG ACA AGG ATC CCG TCG TTT TAC AAC G-3’ (F1) and 5’-TAG AAG GCA CAG TCG AGG C-3’ (R1), followed by ligation into the lentiviral expression vector pLVX-IRES-Puro (Clontech) via the XhoI site. *Plin5* cDNA was amplified from pcDNA4/HisMaxC-mPlin5 using the primers 5’-ATT CCT CGA GCC ACC ATG GAC CAG AGA GGT GAA GAC AC-3’ (F2) and 5’-CGC GGT ACC TCA GAA GTC CAG CTC TGG CAT CAT TG-3’ (R2), and cloned into pEYFP-C1 (Takara Bio USA) via the XhoI and KpnI restriction sites. cDNA encoding FLAG-tagged PLIN5 was amplified from pEYFP-C1-mPlin5 using the primers 5’-ATA GAA TTC CAT GGA TTA CAA GGA CGA CGA TGA CAA GGA CCA GAG AGG TGA AGA CAC-3’ (F3) and 5‘-ATG CGG CCG CTC AGA AGT CCA GCT CTG G-3’ (R3), followed by ligation into pLVX-IRES-Puro via the EcoRI and NotI restriction sites. PLIN5(∆424-463)-encoding cDNA was amplified from pEYFP-C1-mPlin5 using the primers 5’-ATT CCT CGA GCC ACC ATG GAC CAG AGA GGT GAA GAC AC-3’ (F2) and 5’- CGC GGT ACC TCA TGC CCA GCG AGG ATC-3’ (R4), and ligated into pEYFP-C1 via the XhoI and KpnI restriction sites. cDNA encoding FLAG-tagged PLIN5(∆424-463) was amplified from pEYFP-C1-mPlin5 using the primers 5’-ATA GAA TTC CAT GGA TTA CAA GGA CGA CGA TGA CAA GGA CCA GAG AGG TGA AGA CAC-3’ (F3) and 5’-TAG ACT CGA GTC ATG CCC AGC GAG GAT CAG-3’(R5), and cloned into pLVX-IRES-Puro via the EcoRI and XhoI restriction sites. For generation of plasmids encoding additional PLIN5 C-terminal truncation variants, stop codons were inserted into the full length *Plin5* CDS at specific positions using the Q5 site-directed mutagenesis kit (E0554S, NEB) according to the manufacturer’s instructions. The nucleotide sequences of the primer pairs used for site-directed mutagenesis are listed in supplemental Table 1. All generated vector constructs were validated by DNA sequencing (Microsynth Austria GmbH, see supplemental Fig. S2).

**Co-immunoprecipitation experiments**

HEK-293T cells were transfected with vector constructs encoding FLAG-tagged wild type or mutant PLIN5, together with plasmids encoding either Xpress-tagged ATGL, CGI-58 or β-Gal using Metafectene. Twenty-four hours after transfection the cells were washed three times with PBS, harvested by scraping and lysed in ice cold buffer A [50 mM TRIS/HCl pH 7.4, 150 mM NaCl, 1 mM EDTA, 1% (v/v) NP-40, 20 μg/ml leupeptine, 2 μg/ml antipain, 1 μg/ml pepstatin] at 4°C for one hour on a rotating wheel. Cell debris was removed by centrifugation at 1,000 × g and 4°C for 10 minutes. Post nuclear supernatant (PNS) protein concentrations were determined using Pierce BCA reagent (23225, Thermo Fisher Scientific) and BSA as a standard. Next, 20 μl Anti-Flag M2 affinity gel (A2220, Merck Millipore) was prewashed with ice cold buffer A and incubated with equal amounts of lysate proteins (1 mg) at 4°C for 16 hours on a rotating wheel. Subsequently, the agarose beads were washed six times with ice cold buffer A and heated in 2× Laemmli buffer (50 µl) at 95°C for 10 minutes. Input and pulldown fractions were subjected to immunoblot analyses using a mouse anti-Xpress antibody (R910-25, Thermo Fisher Scientific) and an HRP-conjugated rat TrueBlot-ULTRA-anti-mouse Ig secondary antibody (18-8817-33, Rockland Immunochemicals). Thereafter, the membranes were stripped and re-probed using a mouse anti-FLAG M2-HRP antibody (A8592, Sigma-Aldrich). Membrane-bound proteins were stained using Coomassie blue staining solution [45% (v/v) ethanol, 10% (v/v) acetic acid, 0.25% (w/v) Coomassie Brilliant Blue R250], followed by destaining with a solution containing 30% (v/v) ethanol and 10% (v/v) acetic acid.

**Generation of lentiviral particles and establishment of stable cell lines**

HEK-293T cells were cultured in complete medium supplemented with 25 µM chloroquine diphosphate for five hours, followed by polyethylenimine-mediated co-transfection with psPAX2 (Addgene plasmid #12260, kindly provided by Didier Trono) together with pMD2.G (Addgene plasmid #12259, gift from Didier Trono) and pLVX-IRES-Puro constructs encoding recombinant target proteins. After overnight incubation, the culture medium was renewed and cells were cultured for 48 hours. Thereafter, the lentivirus-containing medium was collected, clarified by centrifugation at 500 × g for five minutes, sterile-filtered using cellulose acetate syringe filters with a 0.45 µm pore size (9055503, Bartelt GmbH) and stored at −80°C. For lentiviral transduction, COS-7, AML12 or AC16 cells were seeded in 6-well cell culture plates, followed by incubation in growth medium containing 1 ml lentiviral supernatant and 8 µg/ml polybrene. To enhance transduction efficiency, cells were centrifuged at 1,200 × g and 32°C for one hour (“spinfection”), followed by overnight incubation at 37°C. Subsequently, transduced cells were subjected to positive selection via puromycin treatment. Therefore, the cells were cultured in medium containing puromycin at predetermined suitable concentrations (COS-7 and AML12: 1.5 µg/ml; AC16: 1 µg/ml) for at least ten days, prior to experiments. Mock-transduced control cells died after one to two days of puromycin treatment, whereas lentivirus-infected cells continued to proliferate.

**Mass spectrometry**

*Sample preparation:* Anti-FLAG IP fractions from transgenic AC16 cells were obtained as described in the manuscript (see materials and methods), using 0.1 M glycine-HCL (pH 3.5) to elute bound proteins from the anti-FLAG beads. 50-100 µg of the whole-cell lysates (input fractions) and the total IP fractions were reduced and alkylated with 5 mM TCEP and 20 mM chloroacetamide, respectively. The input samples were then digested with trypsin (1:50 wt/wt) and purified on S-trap mini columns (Protifi), following the manufacturer’s instructions, lyophilized and resuspended in 0.1% formic acid. 200 ng digest was used per injection. The IP fractions were processed using S-trap micro columns following the high recovery protocol and trypsin as a carrier protein, lyophilized and resuspended in 7 µl 0.1% formic acid. 1 µl digest was used per injection.

*Mass spectrometry:* Samples were analysed on timsTOF ion mobility mass spectrometer (Bruker) in-line with UltiMate 3000 UHPLC system (Thermo). Peptides were separated on a reversed-phase C_18_ Aurora column (25 cm × 75 µm) with an integrated CaptiveSpray Emitter (IonOpticks). Mobile phases A and B were 0.1 vol% formic acid in water and 0.1 vol% formic acid in ACN, respectively and the flow rate was 300 nl/min. The fraction of B was linearly increased from 2% to 25% in 90 min, followed by an increase to 40% in 10 min and a further increase to 80% in 10 min and re-equilibration. The spectra were recorded in DIA mode as previously described (4); each time as triplicate for the input samples and a single acquisition for the IPs.

*Data processing:* The DIA data were quantified with DIA-NN (v17.7.16) (5). The spectral library was built from HEK293T whole-cell extract and 144 fractionation samples, and 6 HeLa whole-cell lysate (Pierce) acquisitions using FragPipe (v15.0) ‘SpecLib’ workflow (6, 7) with EasyPQP and UniProt human protein database (downloaded on 12.03.2020) supplemented with the transgene protein sequences. *Data analysis:* The Label-Free Quantification (LFQ) values were log_10_-transformed and averaged, across biological replicates or all technical and biological replicates, for the IPs and the input samples, respectively. After averaging step, the missing values in the IP samples were treated as zeros. The differences between the coIP-ed interactomes were determined by subtracting and centring the resulting delta distributions on zero. The GO term enrichment and protein complex analyses were performed in STRING v11 (8), based on the ranked list of proteins differentially identified in PLIN5wt vs PLIN5(∆461-463) IPs. The mass spectrometry proteomics data have been deposited to the ProteomeXchange Consortium via the PRIDE (9) partner repository with the dataset identifier PXD028541.

**Quantification of the mitochondrial recruitment to LDs**

Quantification of mitochondrial recruitment to LDs was performed in ImageJ. In brief, a 2D Gaussian noise reduction filter (sigma = 1) was applied to two-channel images of acquired LDs (Green C12) and mitochondria (MitoTracker DR). The little background of the images was removed using the “Subtract Background” feature (rolling ball radius = 25). Images of mitochondria were segmented using the implemented Otsu method (ignore black and white activated) and extracted mitochondrial areas stored in the ROI manager. Images of LDs were also binarized using the Otsu method (ignore black and white activated), and the total area of segmented LD structures was measured. Subsequently, binarized mitochondrial areas were overlayed and deleted from binary images of LDs. Afterward, the percentage of the decrease of the total LD area was determined and used as a measure for the overlap of mitochondria and LD. More than 20 cells per cell type were analyzed, whereas the individual images contained a variable number of cells.

**Seahorse XF respirometry of living AC16 cells**

AC16 cells were seeded in a 96-well Seahorse XF cell culture plate that had been precoated with 0.1% gelatin (5,000 cells per well). Following overnight incubation, the culture medium was changed to fresh complete medium, either containing or lacking 400 µM OA-BSA, and the cells were grown to full confluence for 24 hours. Prior to respirometry analysis, the cells were washed with PBS, followed by incubation in serum-free DMEM (D5030, Gibco) containing 5 mM glucose and 2 mM GlutaMAX, pH 7.4, for 60 minutes at 37°C in a 95% humidified non-CO_2_ atmosphere. During this incubation period, a pre-hydrated Seahorse sensor cartridge was loaded with compounds for injection, followed by calibration of the Seahorse XFe96 analyzer (Agilent Technologies). Subsequently, the cellular oxygen consumption rate (OCR) as well as the extracellular acidification rate (ECAR) upon sequential injection of 20 µM forskolin, 1 µM oligomycin A (75351, Sigma-Aldrich), 1 µM carbonyl cyanide 4-(trifluoromethoxy)phenylhydrazone (FCCP, C2920, Sigma-Aldrich) and 1 µM antimycin A (A8674, Sigma-Aldrich) [final concentrations] were determined. For each measuring point, the assay medium was mixed for two minutes, followed by three-minute measurement. Thereafter, the cells were washed three times with PBS and lysed in 0.3 N NaOH and 0.1% (w/v) SDS by shaking for six hours at RT. Protein content was quantified using Pierce BCA reagent. The measured OCR and ECAR were normalized to protein content and standard metabolic parameters including basal respiration, proton leak, ATP-linked respiration, maximal respiration and mitochondrial spare capacity were calculated according to the manufacturer’s instructions (Agilent Technologies). For calculation of the stimulated respiration, the minimal OCR after antimycin A treatment was subtracted from the maximal OCR during forskolin treatment. ATP-linked respiration upon forskolin stimulation was calculated by subtracting the minimal OCR after oligomycin treatment from the maximal OCR after forskolin injection. Mitochondrial spare capacity during forskolin treatment was calculated by subtracting the stimulated OCR from the maximal respiration. To visualize the metabolic shift upon forskolin-treatment, mean OCR was plotted against mean ECAR, using the values determined during the last time points of the basal or forskolin-stimulated conditions. The same values were further used, to calculate the OCR to ECAR ratios, allowing quantification of metabolic shift.

**Subcellular fractionation**

Cytosolic and nuclear fractions of cultured cells were prepared applying the REAP method as previously described (10, 11), using PBS containing 0.5% NP-40, 20 μg/ml leupeptine, 2 μg/ml antipain, 1 μg/ml pepstatin and 1 mM PMSF for cell lysis and washing of nuclear pellets.

Cytosol/membrane fractions as well as mitochondria-enriched fractions were obtained by differential centrifugation. Cells were seeded in cell culture dishes (100 mm), washed three times with PBS and harvested using a cell scraper. Cell pellets were resuspended in 500 µl fractionation buffer (20 mM HEPES pH 7.4, 10 mM KCl, 2 mM MgCl_2_, 1 mM EDTA, 1 mM DTT, 20 mg/ml leupetine, 2 mg/ml antipain, 1 mg/ml pepstatin), incubated for 15 minutes on ice and lysed by passing the cell suspension through a 27-gauge needle ten times. The cell lysate was incubated for 20 minutes on ice. Next, the samples were centrifuged at 750 × g and 4°C for 5 minutes to remove cell debris. The supernatants were centrifuged at 10,000 × g and 4°C for 10 minutes to obtain mitochondria-enriched pellets as well as cytosole/membrane fractions. The mitochondrial pellets were resuspended in 150 µl PBS containing 0.1% (w/v) SDS and briefly sonicated to homogenize the lysate. Protein concentrations of the cellular fractions were determined using Bradford reagent (5000006, Bio-Rad, cytosol/membrane fractions) and the Pierce BCA reagent (mitochondrial fractions).

**Isolation of total DNA and quantification of mitochondrial DNA content**

Total DNA from AC16 cells was isolated using the DNeasy Blood and Tissue Kit (69504, Qiagen) according to the manufacturer’s instructions. The relative copy number of mitochondrial DNA (mtDNA) and nuclear DNA (nDNA) was determined by qPCR according to (12). Therefore, the mitochondrial cytochrome c oxidase (*MTCO1*) and the NADH dehydrogenase flavoprotein 1 (*NDUFV1*) genes were amplified as markers for mtDNA and nDNA, respectively. The used primer pairs were 5’-TCT CAG GCT ACA CCC TAG ACC A-3’ (MTCO1-F) together with 5’-ATC GGG GTA GTC CGA GTA ACG T-3’ (MTCO1-R), or 5’-TGT GTG AGA CGG TGC TGA TGG A-3’ (NDUFV1-F) together with 5’-CGA TGG CTT TCA CGA TGT CCG T-3’ (NDUFV1-R). Expression levels were calculated using the ΔΔCt-method and relative mtDNA copy numbers were analyzed by determining the mtDNA/nDNA ratios.

**Gene expression**

RNA from transgenic AC16 cells was isolated using TRIzol reagent (15596018, Thermo Fisher Scientific), followed by digestion with DNase I (M0303S, NEB). One microgram RNA was reverse-transcribed using the LunaScript RT SuperMix Kit (NEB). qPCR was performed using the Universal SYBR Green Supermix (1725124, Bio-Rad) and the StepOnePlus system (Thermo Fisher Scientific). Relative *PDK4* mRNA levels were quantified via the ΔΔCT method with *36B4* as the reference gene. The used primer pairs were 5'-GGA AGC ATT GAT CCT AAC TGT GA-3' (hPDK4-F) together with 5'-GGT GAG AAG GAA CAT ACA CGA TG-3' (hPDK4-R), or 5'-GCT TCA TTG TGG GAG CAG ACA-3' (36B4-F) together with 5'-CAT GGT GTT CTT GCC CAT CAG-3' (36B4-R).

**Immunoblot analyses**

Immunoblot analyses were performed using a mouse anti-FLAG M2-HRP antibody (A8592, Sigma-Aldrich), a rabbit anti-GAPDH primary antibody (2118S, Cell Signaling Technology), a mouse anti-total-OXPHOS-human antibody cocktail (ab110411, Abcam), a rabbit anti-COX IV antibody (4844S, Cell Signaling) and a rabbit anti-phospho-PKA substrate antibody (9624, Cell Signaling), using adequate HRP-conjugated secondary antibodies. Antibodies that were specifically used for co-immunoprecipitation experiments are described above. HRP-linked IgG was detected using Clarity Western ECL Substrate (1705061, Bio-Rad) and a Chemidoc Touch Imaging System (Bio-Rad). Signal intensities were quantified using Image Lab software (Bio-Rad). Membrane bound proteins were stained using Coomassie blue staining and destaining solution, as described above.

**References**

1 Pollak NM, Jaeger D, Kolleritsch S, Zimmermann R, Zechner R, Lass A *et al.* The interplay of protein kinase a and Perilipin 5 regulates cardiac lipolysis. *J Biol Chem* 2015; **290**: 1295–1306.

2 Pollak NM, Schweiger M, Jaeger D, Kolb D, Kumari M, Schreiber R *et al.* Cardiac-specific overexpression of perilipin 5 provokes severe cardiac steatosis via the formation of a lipolytic barrier. *J Lipid Res* 2013; **54**: 1092–102.

3 Lass A, Zimmermann R, Haemmerle G, Riederer M, Schoiswohl G, Schweiger M *et al.* Adipose triglyceride lipase-mediated lipolysis of cellular fat stores is activated by CGI-58 and defective in Chanarin-Dorfman Syndrome. *Cell Metab* 2006; **3**: 309–319.

4 Meier F, Brunner AD, Frank M, Ha A, Bludau I, Voytik E *et al.* diaPASEF: parallel accumulation–serial fragmentation combined with data-independent acquisition. *Nat Methods* 2020; **17**: 1229–1236.

5 Demichev V, Messner CB, Vernardis SI, Lilley KS, Ralser M. DIA-NN: neural networks and interference correction enable deep proteome coverage in high throughput. *Nat Methods* 2020; **17**: 41–44.

6 Kong AT, Leprevost F V., Avtonomov DM, Mellacheruvu D, Nesvizhskii AI. MSFragger: Ultrafast and comprehensive peptide identification in mass spectrometry-based proteomics. *Nat Methods* 2017; **14**: 513–520.

7 Demichev V, Yu F, Teo GC, Szyrwiel L, Rosenberger GA, Decker J *et al.* High sensitivity dia-PASEF proteomics with DIA-NN and FragPipe. *bioRxiv* 2021; : 2021.03.08.434385.

8 Szklarczyk D, Gable AL, Lyon D, Junge A, Wyder S, Huerta-Cepas J *et al.* STRING v11: Protein-protein association networks with increased coverage, supporting functional discovery in genome-wide experimental datasets. *Nucleic Acids Res* 2019; **47**: D607–D613.

9 Perez-Riverol Y, Csordas A, Bai J, Bernal-Llinares M, Hewapathirana S, Kundu DJ *et al.* The PRIDE database and related tools and resources in 2019: Improving support for quantification data. *Nucleic Acids Res* 2019; **47**: D442–D450.

10 Nabbi A, Riabowol K. Rapid isolation of nuclei from cells in vitro. *Cold Spring Harb Protoc* 2015; **2015**: 769–772.

11 Nabbi A, Riabowol K. Isolation of nuclei. *Cold Spring Harb Protoc* 2015; **2015**: 731–734.

12 Quiros PM, Goyal A, Jha P, Auwerx J. Analysis of mtDNA/nDNA Ratio in Mice. *Curr Protoc Mouse Biol* 2017; **7**: 47–54.
